# HIV-1 binds dynein directly to hijack microtubule transport machinery

**DOI:** 10.1126/sciadv.adn6796

**Published:** 2025-06-18

**Authors:** Somayesadat Badieyan, Drew Lichon, Sevnur Komurlu Keceli, Michael P. Andreas, John P. Gillies, Wang Peng, Jiong Shi, Morgan E. DeSantis, Christopher R. Aiken, Till Böcking, Tobias W. Giessen, Edward M. Campbell, Michael A. Cianfrocco

**Affiliations:** ^1^ Life Sciences Institute, University of Michigan, Ann Arbor, MI, USA.; ^2^ Department of Microbiology and Immunology, Stritch School of Medicine, Loyola University Chicago, Chicago, IL, USA.; ^3^ Department of Biological Chemistry, Michigan Medicine, University of Michigan, Ann Arbor, MI, USA.; ^4^ Department of Molecular, Cellular, and Developmental Biology, College of Literature, Sciences, and the Arts, University of Michigan, Ann Arbor, MI USA.; ^5^ EMBL Australia Node in Single Molecule Science and ARC Centre of Excellence in Advanced Molecular Imaging, School of Medical Sciences, UNSW Sydney, Sydney, New South Wales, Australia.; ^6^ Department of Pathology, Microbiology, and Immunology, Vanderbilt Institute for Infection, Immunology, and Inflammation, Vanderbilt University Medical Center, Nashville, TN, USA.

## Abstract

HIV-1 uses the microtubule cytoskeleton to reach the host cell nucleus during replication, yet the molecular basis for microtubule-dependent HIV-1 motility is poorly understood. Using in vitro reconstitution biochemistry and single-molecule imaging, we found that HIV-1 binds to the retrograde microtubule-associated motor, dynein, directly and not via a cargo adaptor, as has been previously suggested. The HIV-1 capsid lattice binds to accessory chains on dynein’s tail domain. Further, we demonstrate that multiple dynein motors tethered to rigid cargoes, such as HIV-1 capsids, display reduced motility, distinct from the behavior of multiple motors on membranous cargoes. Our results provide an updated model of HIV-1 trafficking wherein HIV-1 binds to dynein directly to “hijack” the dynein transport machinery for microtubule motility.

## Teaser

HIV-1 is an opportunistic hijacker of microtubule machinery.

## INTRODUCTION

Because of the crowded nature and large size of eukaryotic cells, diverse processes, including organelle trafficking, mRNA localization, and cell division, take advantage of microtubule-based trafficking systems (*1*). The directional cargo transport along microtubules is accomplished by the actions of opposite-directed motor proteins kinesin (*2*) and dynein (*3*).

The size of viral particles and the high density of the cytoplasm force viral pathogens to evolve mechanisms for hijacking and manipulating host transport machinery to navigate the host cell and support their replication (*1*, *4*). One example is the HIV-1 (*5*). Nearly 20 years ago, live-cell imaging (*6*, *7*) showed that HIV-1 exploits the microtubule network to move throughout the cell. Kinesin-1 or dynein motor protein depletion inhibits HIV-1 trafficking in the cytoplasm, nuclear translocation, uncoating, and infection (*8*, *9*), showing that microtubule-associated motors play an integral part in the HIV-1 life cycle.

Despite this previous work demonstrating that HIV-1 hijacked the microtubule cytoskeleton during replication, the molecular basis for the interaction remains poorly defined. For instance, a suite of perturbations showed that dynein is required for motility but failed to point to a mechanism of attachment: (i) Microinjection of an inhibitory antibody against the dynein intermediate chain attenuates HIV-1 infection and its retrograde transport (*6*); (ii) knockdown of dynein light chain 1 (*10*) or light chain 2 (*11*) reduces HIV-1 integrase transport or completion of reverse transcription, respectively; and (iii) knockdown of dynein heavy chain resulted in reduced retrograde movement of the HIV-1, viral infection, and nuclear entry (*8*, *12*). Furthermore, probing with antibodies specific to dynein heavy chain and dynein intermediate chain revealed that endogenous dynein in cell extracts can bind to the HIV-1 capsid in vitro (*12*). Thus, while multiple subunits of the dynein complex are required for HIV-1 infection, cell-based experiments could not map the molecular mechanism of trafficking.

Here, we sought to understand how HIV-1 exploits the dynein motor for motility using a purified, reconstituted system that would allow us to visualize microtubule-dependent HIV-1 trafficking in vitro. We found that HIV-1 binds to the dynein motor directly instead of using a cargo adaptor such as Bicaudal D cargo adaptor 2 (BicD2). The direct attachment of HIV-1 to dynein allows HIV-1 to leverage microtubule-driven motility of that many divergent dynein cargo adaptors, suggesting that HIV-1 opportunistically traffics with different cargoes in the cell. We show that HIV-1 capsid recruits dynein by binding to the intermediate chain bound to light chains located within dynein’s tail. Last, our work suggests that HIV-1 uses a distinct region of the lattice to bind dynein, which does not overlap with known binding factors. Our results reveal that the direct binding of HIV-1 capsid to dynein allows the virus to hijack multiple active motors and establishes how HIV-1 exploits microtubule motor proteins to support infection.

## RESULTS

### HIV-1 cores bind directly to dynein for microtubule recruitment

**Fig. 1.**
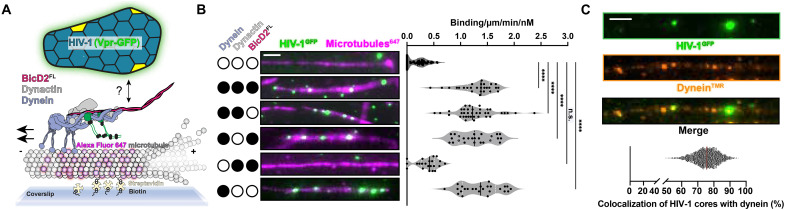
HIV-1 cores bind to dynein directly for microtubule recruitment in vitro. (**A**) Schematic of HIV-1 microtubule motility assay. The dynein motor assembles with dynactin and BicD2 for motility. (**B**) Microtubule recruitment assay and quantification of HIV-1 (GFP-Vpr) with dynein, dynactin, and BicD2^FL^. (**C**) Colocalization of HIV-1^GFP^ with fluorescently labeled TMR-labeled dynein (dynein^TMR^). Dynein was kept at 10 nM. Scale bars, 5 μm. ****P ≤ 0.0001 and “n.s.” represents *P* = 0.3991.

Previous work suggested that BicD2, a dynein cargo adaptor, binds to the HIV-1 capsid or nucleocapsid in vitro (*12*, *13*), which has led to the model that full-length BicD2 (BicD2^FL^) is required for the microtubule-based trafficking of HIV-1 (Fig. 1A). When not bound to cargo, BicD2^FL^ adopts an autoinhibited conformation involving an intramolecular interaction that prevents dynein binding (*14*–*17*). On the basis of the proposed HIV-1 trafficking model, the interaction of the BicD2 cargo-binding domain (coiled-coil 3) with the HIV-1 capsid releases BicD2^FL^ autoinhibition (fig. S1A), allowing the N terminus of BicD2 to interact with dynein in a manner analogous to established BicD2^FL^ binding partners, including Ras-related protein Rab-6 (*18*), Egalitarian mRNA (*19*), and nucleoporin 358 or Nup358 (*16*).

To directly test this model, we combined purified HIV-1 cores [fluorescently labeled with green fluorescent protein (GFP)–(Viral Protein R) (Vpr)] with purified dynein, dynactin, and BicD2^FL^. HIV-1 cores are conical capsid protein (CA) surrounding an internal ribonucleoprotein complex that is competent for reverse transcription and subsequent DNA integration (*20*, *21*). Previous work demonstrated that HIV-1 cores with GFP-Vpr incorporated allow visualization of single-core trafficking in cells (*6*, *22*, *23*). After confirming the integrity of purified HIV-1 (GFP-Vpr) cores (fig. S1B), we measured HIV-1 core recruitment to microtubules adhered to a coverslip via single-molecule total internal reflection fluorescence (TIRF) microscopy assays (Fig. 1A). TIRF microscopy showed that the presence of dynein-dynactin-BicD2^FL^ increased HIV-1 cores binding to microtubules by ~3.4 times (Fig. 1B). Unexpectedly, however, despite visualizing HIV-1 recruitment to microtubules, the HIV-1 cores did not undergo active motility (fig. S1C).

Given our negative result, we wanted to confirm that the reagents in our assay were properly functioning. First, we tested dynein activity by adding dynactin and a constitutively active truncation of BicD2, BicD2^1–598^. As expected (*24*, *25*), the complexes showed robust activity (fig. S2A). Next, to confirm the activity of BicD2^FL^, we added a cargo peptide from Nup358, Nup358^min-zip^. Nup358^min-zip^ is an α helix (residues 2162 to 2184) from Nup358 engineered to dimerize by a GCN4 (general control non-derepressible 4) leucine zipper that is capable of being trafficked in a complex of dynein-dynactin-BicD2-Nup358^min-zip^ (*16*). Using our reconstituted single-molecule assay, we demonstrated that fluorescently labeled Nup358^min-zip^ can be recruited to microtubules in the presence of dynein, dynactin, and BicD2^FL^ (fig. S1D). Excluding either BicD2 or dynein reduces Nup358^min-zip^ recruitment by approximately 15 times (fig. S1D). In addition, Nup358^min-zip^ was processively transported in the presence of dynein, dynactin, and BicD2 (fig. S1, E and F). Together, these experiments demonstrate that the dynein, dynactin, and BicD2^FL^ are active, suggesting that HIV-1 does not activate the motility of dynein via BicD2^FL^.

In another attempt to test our reconstituted system, we designed drop-out experiments using single-molecule TIRF microscopy to determine the minimal components needed for HIV-1 microtubule recruitment. Unexpectedly, removing BicD2^FL^ and/or dynactin from the imaging reaction did not affect HIV-1 core recruitment to microtubules (Fig. 1B and fig. S1C). In contrast, removing dynein showed a marked loss of HIV-1 core microtubule recruitment (Fig. 1B and fig. S1C). These data indicate that HIV-1 cores require dynein to bind microtubules, and this interaction occurs in the absence of the cargo adaptor BicD2^FL^. To directly visualize the interaction, we imaged HIV-1 cores with fluorescently labeled dynein and saw that 75.6% of microtubule-bound HIV-1 cores are colocalized with fluorescently labeled dynein (Fig. 1C). Thus, HIV-1 binds to dynein directly for microtubule recruitment in vitro.

### HIV-1 exploits multiple dynein-activating adaptors for motility

**Fig. 2.**
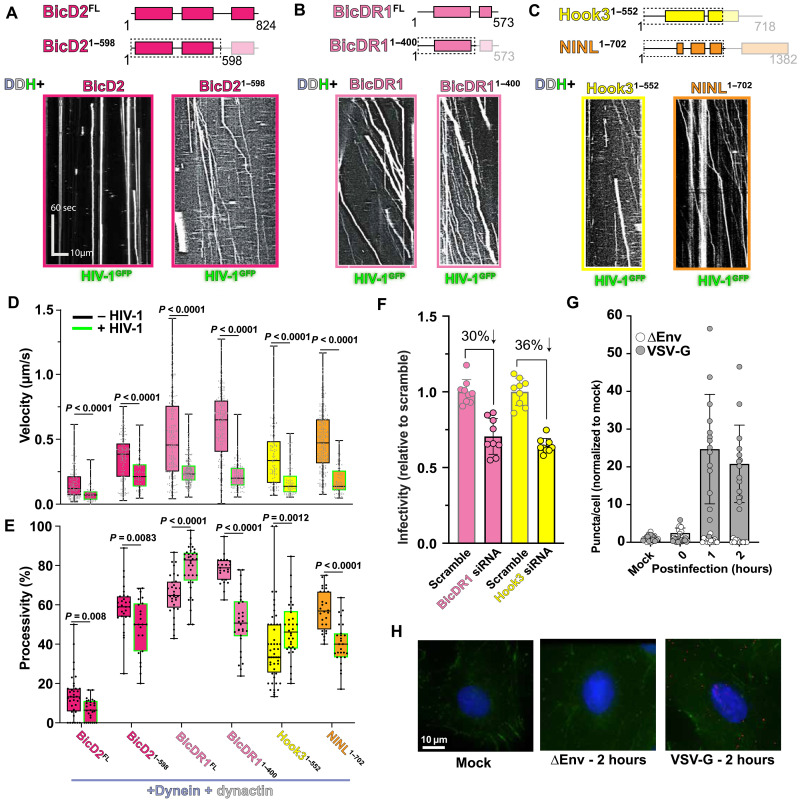
HIV-1 exploits multiple dynein-activating adaptors for in vitro motility. Schematic coiled-coil presentation of BicD2 (**A**), BicDR1 (**B**), Hook3, and NINL (**C**) constructs shown alongside reconstitution of HIV-1 motility with dynein-dynactin (DDH) and adaptor. (**D**) Quantification of velocity for dynein-dynactin-adaptor and HIV-1 cores. (**E**) Quantification of processivity for dynein-dynactin-adaptor and HIV-1 cores. (**F**) HIV-1 infection assays using luciferase reporter for small interfering RNA (siRNA)–mediated knockdowns of BicDR1 and Hook3 in A549 cell. (**G** and **H**) Proximity ligation assay (PLA) between HIV-1 CA (P24) and BicDR1 for uninfected (mock), negative control HIV-1 strain that cannot enter cells (∆Env), and HIV-1 pseudotyped with vesicular stomatitis virus glycoprotein (VSVG) Env protein. (G) Quantification of puncta per cell. (H) Representative fluorescence images for 2 hours postinfection.

Our results suggest that dynein-dynactin-BicD2^FL^ does not support the motility of HIV-1 because HIV-1 binds to dynein directly instead of binding and activating BicD2^FL^. This model predicts that dynein activation is independent of HIV-1 binding. To test this, we compared HIV-1 motility with dynein and dynactin using either BicD2^FL^ or a C-terminal truncation BicD2 that is missing the autoinhibitory cargo-binding domain and constitutively activates dynein motility (Fig. 2A). We found that dynein-dynactin incubated with BicD2^1–598^ drove the processive motility of HIV-1 cores (Fig. 2A). These results indicate that the BicD2 C-terminal cargo-binding domain is not needed for HIV-1 binding to microtubules or motility, as previously proposed (*12*, *13*).

Considering that BicD2^1–598^ activates HIV-1 motility without a cargo-binding domain, we hypothesized that any cargo adaptor promoting dynein motility would enable HIV-1 motility. To test this, we incubated dynein and dynactin with several cargo adaptor constructs previously shown to activate dynein motility in vitro and asked whether the resulting complexes could generate processive HIV-1 motility. Here, we used full-length and truncated versions of a BicD2 ortholog, which is not autoinhibited, called BicDR1 (BicDR1^FL^ and BicDR1^1–400^) (*26*, *27*), and two well-characterized truncations of Hook3 (Hook3^1–552^) (*28*, *29*) and Ninein-like (NINL^1–702^) (*30*) (fig. S2, A and B). None of these cargo adaptors have previously been implicated in HIV-1 trafficking or infection. Unexpectedly, we observed processive motility of HIV-1 with all nonautoinhibited cargo adaptor constructs tested (Fig. 2, A to C). This finding supports our model that dynein-mediated HIV-1 motility does not require HIV-1 binding to adaptors as cargo. In addition, we observed almost the same landing rate of HIV-1 on microtubules with all adaptors, which suggests that the identity of the cargo adaptors does not alter the basal association of HIV-1 and dynein (fig. S2C).

Comparison of dynein-driven HIV-1 movement versus dynein-adaptor movement showed that dynein-driven HIV-1 motility is significantly slower than the motility of dynein alone (Fig. 2D). Analysis of the velocity of HIV-1 bound to all cargo adaptors showed an average velocity of ~0.16 μm/s, which is 2× to 4× slower than the overall velocity for dynein-dynactin with the same cargo adaptors (without HIV-1 cargo) (Fig. 2D). Thus, the velocity of HIV-1 bound to dynein in vitro is slower than other dynein-adaptor or dynein cargo complexes.

While we did not observe a significant difference in the velocity of HIV-1 being trafficked by dynein-dynactin complexed to different cargo adaptors, we did observe a difference in processivity with various adaptors (Fig. 2E). Over 80% of the HIV-1 cores that were recruited to the microtubule move processively in the presence of dynein-dynactin-BicDR1^1–598^, whereas this was reduced to ~50% and less for the N-terminal end of BicDR1, BicD2, Hook3, and NINL. Last, <10% of the HIV-1 recruited to the microtubule via dynein bound to BicD2^FL^ was processive. The observed processivity trends are directly correlated to the intrinsic ability of each cargo adaptor to activate dynein-dynactin in the absence of HIV-1, showing that HIV-1 does not affect the processivity of dynein activated by divergent cargo adaptors (Fig. 2E).

Our model suggests that any cargo adaptor that enables dynein motility can function as a host factor that supports HIV-1 infection. To test this model, we depleted BicDR1 and Hook3 cargo adaptors from A549 cells and monitored infectivity (fig. S3, A to E). We did not test NINL in the infectivity assays because NINL contributes to innate immunity via interferon signaling (*31*). In support of our updated model of HIV-1 motility, the knockdown of BicDR1 or Hook3 in A549 cell lines reduced HIV-1 infectivity by nearly 30 and 36%, respectively (Fig. 2F and fig. S3, A to E), whereas infectivity was reduced by 42% for BicDR1-depleted CHME-5 cell lines (fig. S3, F and G). We observed, within HeLa TZM-bl cells, no change in HIV-1 infectivity following the knockdown of either BicDR1 or Hook3 (fig. S3, H and I). These results recapitulate previous studies and suggest a cell type–specific utilization of dynein-activating adaptors (*12*, *13*).

Host factors that support infection should also colocalize with HIV-1. Using proximity ligation assay (PLA) to monitor the proximity of HIV-1 capsid with BicDR1 in A549 cells, we found an ~80× increase in HIV-1–BicDR1 colocalization 1 to 2 hours postinfection (Fig. 2G and fig. S3J). This is comparable to the colocalization previously observed with BicD2 (*13*). Together, our work suggests that HIV-1 exploits multiple dynein cargo adaptors to support infection.

### The HIV-1 capsid is sufficient for dynein-directed motility

**Fig. 3.**
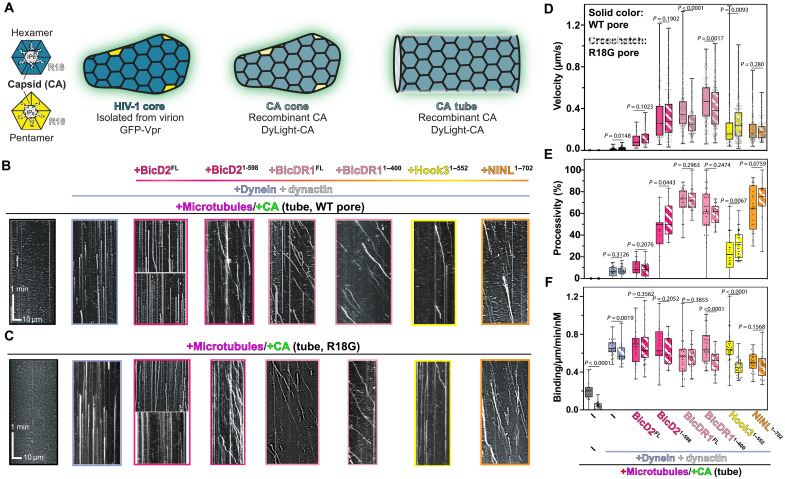
HIV-1 capsid is sufficient for dynein-directed motility in vitro. (**A**) Structural models of CA hexamers and pentamers shown alongside HIV-1 cores, CA tubes (formed with high salt or A92E/A204C), or CA cones [formed by inositol hexakisphosphate (IP_6_) or A92E/A204C]. (**B** and **C**) Microtubule recruitment and motility reconstitution of CA particles with WT pore (A92E/A204C) and CA particles with mutated pores (A92E/A204C/R18G) in presence of dynein, dynactin, and different activators. Quantification of velocity (**D**) processivity (**E**), and recruitment (**F**) for HIV-1 tubes related to motility assays at (B) and (C).

After observing that HIV-1 cores can bind to dynein and undergo directional motility, we hypothesized that dynein is binding to the viral capsid shell of the cores. The capsid shell surrounding cores binds to numerous host factors (*32*). Whether the capsid remains largely intact until it docks at the nuclear pore (*33*–*35*) or begins disassembling in the cytoplasm (*36*, *37*), it can serve as a critical platform for interactions with dynein. To test whether the CA can bind dynein directly, we performed TIRF motility assays using cross-linked, fluorescently labeled capsid particles produced by self-assembly of recombinant CA (A92E/A204C) under conditions that yield a mixture of capped tubes and cones from building blocks of CA hexamers and pentamers (Fig. 3A and fig. S4A). We saw that, similar to HIV-1 cores, dynein alone recruits CA particles to microtubules and BicD2^FL^ is unable to activate motility (Fig. 3B). When constitutively active cargo adaptors were included in the assay, we observed robust motility for all adaptors tested (BicD2^1–598^, BICDR1^FL^, BicDR1^1–400^, HOOK3^1–552^, and NINL^1–702^) (Fig. 3B). Again, similar to HIV-1 cores, we see that the velocity of moving CA particles is reduced compared to dynein not loaded with viral cargo (Fig. 3, D and E). In addition, the overall dynein-dependent microtubule-binding rate of CA particles is similar to that observed with HIV-1 cores (Fig. 3F). On the basis of these results, the intact HIV-1 capsid lattice is sufficient for binding motile dynein in vitro.

Three well-established and discrete sites on the HIV-1 capsid lattice are the central pore of the hexamer, the FG (phenylalanine-glycine)-binding pocket, and the cyclophilin A (CypA)–binding loop (fig. S4B). Given that each site interacts with multiple, unique host factors, we speculated that dynein may engage with the viral capsid via one of these sites.

First, we examined whether dynein interacts with the central pore, which lies at the center of CA hexamers and is lined by six positively charged arginine residues (R18) (Fig. 3A and fig. S4B). The R18 pore binds to nucleotides (*38*) and inositol hexakisphosphate (IP_6_) (*39*, *40*), where the latter plays a critical role in HIV-1 capsid maturation (*39*, *40*). Recent work also showed that the kinesin-1 adaptor, fasciculation and elongation protein zeta 1 (FEZ1), binds the central pore of hexamers (*41*). To test whether dynein binds to the R18 pore, we purified a mutant CA particle with glycine introduced into the pore (R18G). In TIRF assays, capsid particles assembled from R18G CA (fig. S4A) were recruited to microtubules and transported similarly to capsid particles with R18 (Fig. 3C), with nearly the same velocity (Fig. 3D), processivity (Fig. 3E), and binding rate (Fig. 3F) as wild type (WT). The WT pore capsid, however, showed a slightly higher binding rate to the microtubules even in the absence of dynein (Fig. 3F). We reason that this is due to a partial electrostatic interaction of the positively charged pores in WT with the negatively charged surface of microtubules. In addition, IP_6_ up to 100 μM (which should act as a competitive inhibitor to any factor that binds to the R18 pore) did not affect the microtubule association, processivity, or velocity of capsid particles (fig. S4D). From these data, we conclude that HIV-1 does not bind dynein using the central pore.

Next, we examined whether dynein binds to the FG-binding pocket. Small molecules such as GS-6207 and PF74, along with host factors, such as Nup153 and cleavage and polyadenylation specificity factor 6 (CPSF6), bind at the FG-binding pocket (fig. S4B) (*42*). Monitoring dynein, dynactin, and BicDR1 (DDR)–mediated trafficking of HIV-1 cone-shaped capsid (fig. S4C) in the presence of PF74 showed that excess PF74 did not affect HIV-1 capsid microtubule binding, processivity, or velocity (fig. S4D). Moreover, mutating CA N57 within the FG-binding pocket (fig. S4B) significantly impairs the binding of both CPSF6 and Nup153 (*43*). Despite this, motility parameters of the DDR-mediated transport of the assembled HIV-1 capsid bearing the N57D mutation were not adversely affected (fig. S4E).

Last, we wanted to determine whether the CypA-binding loop was involved in the dynein-mediated recruitment of HIV-1 to microtubules. Given the low affinity of CypA for the HIV-1 capsid [dissociation constant (*K*_d_) > 13 μM) (*44*), performing a competition assay was not feasible. Instead, we analyzed the dynein-mediated recruitment and movement of the G89V capsid. This mutation in the CypA-binding loop (fig. S4B) completely abolishes the HIV-1 CypA binding to the capsid (*45*). However, in our single-molecule experiments, this mutation did not affect either the binding or trafficking of the capsid (fig. S4E).

Together, these results suggest that dynein does not bind to the central pore, FG-binding pocket, or CypA loop of HIV-1 capsid. The fully WT capsid assembled by IP_6_ showed reduced dynein-mediated recruitment to microtubules compared to the cross-linked capsid, which we have used in all experiments (fig. S4E). However, the number of processive events and the velocity of WT capsid did not change significantly from those of the cross-linked capsid bearing A92E/A204C mutations (fig. S4E). Although we currently do not have a clear explanation for this reduced binding, we believe that the A92E/A204C mutations should not play a role in this regard, as the purified WT cores were still capable of being recruited to microtubules in a dynein-based manner (Figs. 1 and 2).

### HIV-1 capsid binds to the tail domain of dynein

**Fig. 4.**
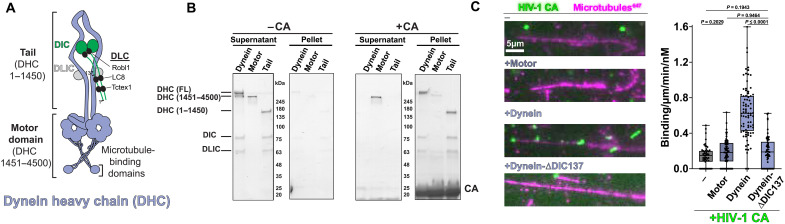
The HIV-1 capsid binds to the dynein tail domain. (**A**) Schematic of dynein structure and segments. (**B**) Copelleting of CA tubes (14C/45C) with dynein, dynein motor, and dynein tail. (**C**) HIV-1 microtubule recruitment assay using the motor domain, full-length dynein, and dynein-∆DIC137 with CA tubes (A92E/A204C capsid).

Human cytoplasmic dynein is a ∼1.4 MDa dimeric complex consisting of a heavy chain (DHC, ∼530 kDa) and several associated subunits: the intermediate chain (DIC), the light intermediate chain (DLIC), and three light chains (DLCs), Rob1/LC7, LC8, and Tctex1 (Fig. 4A). The C-terminal two-thirds of the heavy chain form the adenosine 5′-triphosphate (ATP)–driven motor domain (∼380 kDa) while N-terminal part of the heavy chain associates with DIC and DLIC, where the DLCs bind to the DIC subunit (*3*, *46*). To determine which regions or subunits of dynein contribute to HIV-1 binding, we generated multiple constructs of dynein that have specific regions truncated or accessory chains deleted and monitored the ability of HIV-1 to engage with each construct.

First, we tested whether cross-linked HIV-1 CA tubes (A14C/E45C) bind dynein’s tail or motor domain. To do this, we purified two distinct constructs of dynein: one that consisted of only the tail region (DHC N terminus, DIC, DLIC, and DLCs) (*26*) and another construct that contained only the adenosine triphosphatase (ATPase) motor domain (DHC, 1451 to 4500) (fig. S5, A and B). Using these two constructs, we performed a cosedimentation experiment with CA tubes in vitro. When copelleted with cross-linked CA tubes (A14C/E45C), we saw robust binding for full-length dynein and the tail, whereas the motor domain showed little binding (Fig. 4B). Thus, capsid binds dynein’s tail and not the ATPase motor domains.

To confirm the cosedimentation results and extend our binding data, we used HIV-1 microtubule recruitment assays with dynein truncation constructions. Here, we compared HIV-1 CA tube microtubule recruitment using full-length dynein, motor domain only (DHC, 1451 to 4500), and a mutant form of dynein where the N-terminal 137 residues of DIC are removed, which leads to the loss of light chains LC8 and TcTex1 (dynein-ΔDIC137). Previous work has implicated dynein light chain 1 (LC8) as a factor that is required for HIV-1 replication (*11*), and, in some other retroviruses, LC8 interacts with the capsids (*47*, *48*). After confirming that LC8 and TcTex1 were missing from the dynein-ΔDIC137 complex (fig. S5, C and D), we performed negative stain electron microscopy (EM) to confirm the complex is properly formed (fig. S5E) and then showed that they bound microtubules in a comparable manner to WT dynein (fig. S5F). Next, we monitored dynein-mediated microtubule recruitment of fluorescent CA particles (A92E/A204C) with each dynein construct. Consistent with the sedimentation data generated with CA tubes (A14C/E45C), we observed that the motor domain only (DHC, 1451 to 4500) did not recruit HIV-1 CA particles to microtubules. In contrast, full-length dynein recruited CA particles to microtubules, confirming the cosedimentation data (Fig. 4C). We also observed a marked loss in CA particle recruitment to microtubules when we used dynein-ΔDIC137 (Fig. 4C).

Our data indicated that DIC and light chains LC8 and TcTex1 bind to the HIV-1 capsid. To test this directly, we purified recombinant LC8 and Tctex1 and performed cosedimentation experiments with HIV-1 CA tubes (A14C/E45C) (fig. S5G). In addition to testing the binding of light chains, we also included CypA as a positive control for capsid binding. The binding experiment revealed that LC8 can bind and copellet with HIV-1 CA tubes (A14C/E45C) (fig. S5G).

Together, our biochemical interaction data suggest that the HIV-1 CA lattice binds to dynein’s tail domain using the N terminus of the dynein intermediate chain with LC8 and TcTex1. Future structural work is needed to develop a higher-resolution model of the interaction.

### HIV-1 recruits a team of dynein motors for motility

**Fig. 5.**
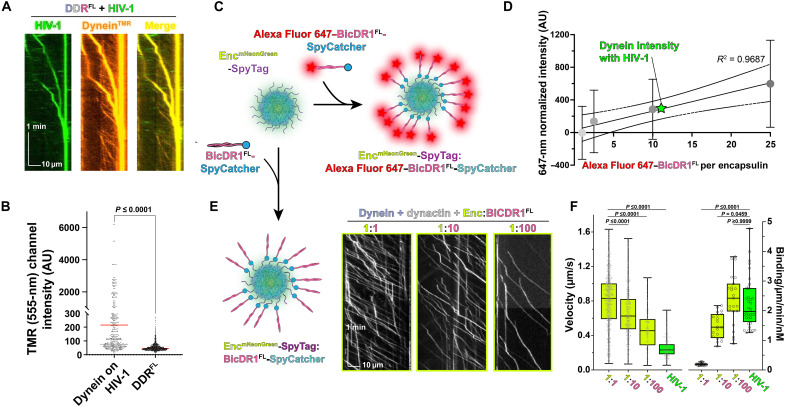
HIV-1 recruits a team of dynein motors for motility. (**A**) Representative kymographs of the colocalization of HIV-1 cores (Vpr-GFP) and TMR-labeled dynein in moving DDR^FL^–HIV-1 complexes. Each channel is shown separately (left and middle) and merged (right). (**B**) Related to (A), fluorescence intensity of TMR-555–labeled dynein in single DDR^FL^ versus colocalized with HIV-1. (**C**) Schematic design of encapsulin-SpyTag fluorescently labeled with SNAP-BicDR1^FL^-SpyCatcher at increasing ratios of BicDR1^FL^:encapsulin. (**D**) Comparison of BicDR1^FL^:encapsulin fluorescence standard curve versus fluorescent signal of dynein on HIV-1 cores. AU, arbitrary units. (**E**) Formation of BicDR1^FL^-SpyCatcher–labeled encapsulin complexes and representative kymographs of encapsulin-mNeonGreen signal with different ratios of BicDR1 in the presence of dynein and dynactin. (**F**) Quantification of velocities and binding rates for encapsulin complexes at each BicDR1 ratio shown alongside data of HIV-1 core with dynein, dynactin, and BicDR1.

Throughout our studies, we observed significantly reduced velocities of motile HIV-1 compared to the velocity of the dynein–dynactin–cargo adaptor alone (Fig. 2D). Considering that the velocities we observed are generally consistent with the average speed of HIV-1 within cells, ~0.2 μm/s (*7*, *13*), we wanted to explore what might cause the slowed velocity of HIV-1 bound dynein complexes. The first experiment to test this monitored HIV-1 motility in the presence of TMR (tetramethylrhodamine)-labeled dynein and compared fluorescence intensities for individually walking dynein-dynactin-BicDR1^FL^ motors versus dynein colocalized with HIV-1 cores (Fig. 5A). Our analysis showed that the dynein fluorescence was, on average, 7.2× brighter for dyneins associated with HIV-1 motility (Fig. 5B). Thus, moving HIV-1 recruits multiple dynein motors.

To quantify how many dynein motors are bound to HIV-1 cores, we compared dynein fluorescence to a standard curve generated using engineered encapsulins. Encapsulins are customizable bacterial nanocompartments that can be exploited for biophysical assays, such as genetically encoded multimeric nanoparticles (*49*). The encapsulin construct used in this study assembles into a *T* = 4 (240 protomers, 42 nm in diameter) icosahedral protein cage (*50*), encapsulating mNeonGreen via an N-terminal fusion to its natively loaded cargo protein, iron-mineralizing encapsulin-associated firmicute(IMEF) (*51*, *52*). Each encapsulin subunit has a surface-exposed C-terminal SpyTag that can efficiently form a covalent bond with its 10-kDa partner protein SpyCatcher (fig. S6, A and B) (*53*).

BicDR1^FL^-SpyCatcher, fluorescently labeled with Alexa Flour 647 on its SNAP-tag (self-labeling protein), SNAP^Alexa647^-BicDR1^FL^-SpyCatcher, was mixed with encapsulin-SpyTag at specific ratios to create decorated encapsulins with 1, 2.5, 10, 25, or 100 SNAP^Alexa647^-BicDR1^FL^-SpyCatcher proteins per encapsulin (Fig. 5C and fig. S6, B and C). The fluorescence intensity of each SNAP^Alexa647^-BicDR1^FL^-SpyCatcher:encapsulin-SpyTag was measured by TIRF on an untreated coverslip (fig. S6D). A standard curve was generated by plotting the mean fluorescence emission of each construct against the number of dyes on each encapsulin (SNAP^Alexa647^-BicDR1^FL^-SpyCatcher) (fig. S6, F and G). In parallel, SNAP-dynein was labeled with the same Alexa Fluor 647 dye and allowed to bind to HIV-1 cores at saturating concentrations. SNAP^Alexa647^-dynein–bound HIV-1 complexes were adhered to untreated coverslips and imaged using 647-nm excitation (fig. S6E), and the average dynein intensity was calculated from dyneins colocalized with HIV-1 cores (fig. S6E). After comparing dynein intensity to the encapsulin standard curve, we see that 11.58 ± 11.27 dynein dimers bind per HIV-1 core. Thus, HIV-1 cores bind multiple dynein motor proteins per core, and our data indicate that the number of dyneins per HIV-1 is not specific but instead varies.

We hypothesized that the reduced velocity we observed was potentially due to the negative interference among multiple dyneins bound to the surface of the HIV-1 capsid. It is possible that collective yet uncoordinated stepping would result in a reduction in velocity (*54*). The observed slowed velocities for HIV-1 were unexpected, given that membranous cargoes such as phagosomes and endosomes recruit 10 to 15 dynein motors in cells and still move with a relatively fast velocity (2 μm/s) (*55*). We speculated that the rigidness of the cargo could cause a difference in dynein behavior on membranes versus HIV-1. While previous work suggested that HIV-1 capsid can undergo breathing (*56*), the flexibility of freely diffusing dynein motors on membranous cargoes represents a much different dynein attachment arrangement than dynein bound to HIV-1 capsid.

To determine whether a capsid-like, rigid cargo prevents the coordinated stepping of multiple dynein motors, we used the synthetic encapsulin-SpyTag cargo. To do this, we bound increasing numbers of BicDR1^FL^-SpyCatcher to assembled encapsulin-SpyTag and imaged the complexes using the incorporated mNeonGreen signal within the encapsulin in the presence of saturating concentrations of dynein and dynactin (Fig. 5E). We found that increasing the ratio of BicDR1^FL^-SpyCatcher per encapsulin (1:1 → 1:10 → 1:100) decreased the overall velocity while increasing the encapsulin complexes’ landing rate (Fig. 5F). When comparing the landing rate and velocity of the 1:100 encapsulin:BicDR1 ratio to HIV-1, we see that HIV-1 capsids exhibited a reduced velocity and a comparable landing rate (Fig. 5F). This result indicates that rigid cargoes such as encapsulin or HIV-1 capsid with multiple bound dyneins may have reduced velocities and increased landing rates compared to membranous cargo.

## DISCUSSION

Here, we used in vitro reconstitution to find that HIV-1 directly binds dynein to hijack the microtubule motility transport machinery. Unexpectedly, we see that dynein binds via its tail domain to the HIV-1 capsid and separately recruits an adaptor protein for activation, allowing HIV-1 to use multiple dynein cargo adaptors for motility. Last, we show that HIV-1 displays reduced velocity compared to native cargoes because teams of dynein motors recruited to the capsid display negative interference with respect to velocity.

**Fig. 6.**
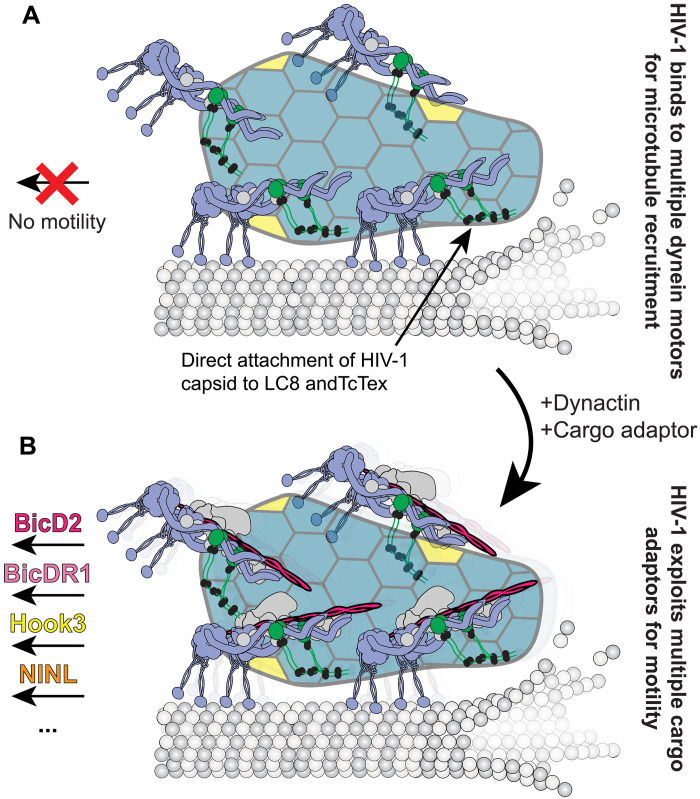
Model of HIV-1 microtubule trafficking by the dynein machinery. (**A**) HIV-1 recruitment to microtubule via multiple dynein motor proteins. The capsid of HIV-1 binds to the tail domain of dynein using the composite binding site of DIC-LC8-TcTex1. When bound to dynein alone, the HIV-1:dynein complexes are immotile. (**B**) HIV-1 achieves microtubule motility by exploiting active dynein cargo adaptors in conjunction with the dynactin core regulator.

Our work establishes an updated framework to understand HIV-1 motility, showing that HIV-1 can bind to motor proteins directly to hijack host cell transport machinery (Fig. 6). In this model, the HIV-1 capsid lattice contacts dynein via light chains in dynein’s tail domain, allowing HIV-1 capsid to be recruited to microtubules (Fig. 6A). Currently, our work does not identify where on capsid dynein binds, despite probing the central pore of the capsid hexamer, the CypA loop, or nuclear pore FG-binding site. Further structural work is needed to determine where dynein binds to HIV-1. Beyond microtubule recruitment, our work shows that a diverse set of constitutively active dynein cargo adaptors activate HIV-1 motility (Fig. 6B). In our model, HIV-1 remains stably bound to dynein’s tail, while the cargo adaptors enable dynein to begin microtubule motility via the combined action of the adaptor and dynactin. Considering the slowed motility of HIV-1:dynein complexes versus dynein complexes alone, the direct attachment of HIV-1 to dynein may negatively affect the ability of dynein to take process steps, although further work is needed to understand if this happens.

We believe the term hijack is appropriate for HIV-1 microtubule transport, given that binding to dynein offers HIV-1 capsid a direct route through the cytoplasm. Given the numerous locations in the cell where dynein is localized (e.g., plasma membrane, organelles, centrosome, or nuclear pores), binding to dynein instead of cargo adaptors allows HIV-1 a flexible mode to attach to microtubules and undergo retrograde trafficking. Co-opting the dynein machinery for transport gives direct transport to HIV-1 instead of undergoing random diffusion through the cytoplasm.

Our reconstituted trafficking system of HIV-1 with the dynein machinery allowed us to perform several experiments to confirm the specificity of the interaction between HIV-1 and dynein. First, we demonstrated that the HIV-1 capsid is sufficient for binding to dynein and for dynein motility (Fig. 3). Next, we used large truncations of dynein to show that removal of the dynein’s tail led to the loss of HIV-1 capsid recruitment to the microtubule (Fig. 4). Notably, the motor domain only construct of dynein (i) binds microtubules and (ii) comprises 3049 amino acid residues, indicating that most of the dynein heavy chain remains in our system. Further, we showed that removing ~5% of the dynein motor via dynein-ΔDIC137, which was a truncation of DIC that no longer binds to LC8 and TcTex1, led to the loss HIV-1 capsid microtubule recruitment (Fig. 4C), although we cannot fully rule out the possibility that other segments of dynein may also contribute to the interaction with the HIV-1 core. Together, these data argue that the interaction of HIV-1 on dynein is specific. Future structural studies will provide more detailed information about this interaction.

Despite this work advancing our understanding of HIV-1 motility, there are several limitations that we want to highlight. First, our reconstituted system from purified components is missing many components from the cellular milieu that may affect the ability of dynein to trafficking HIV-1. For instance, HIV-1 motility is bidirectional in cells (*9*, *57*), suggesting that kinesin-1 is a part of active microtubule trafficking complexes. Since our reconstituted system lacks kinesin, we do not know how kinesin-1 or the kinesin-1 adaptor FEZ1 may affect dynein binding by HIV-1 capsid. Second, without a structural model of the dynein:HIV-1 interaction with and without cargo adaptors and dynactin, we do not know whether the nature of the HIV-1 interaction with the dynein machinery is different depending on whether it is immotile (via dynein alone) or motile (via dynein–dynactin–cargo adaptor) and the exact nature of how light chains facilitate dynein binding to HIV-1 capsid. Last, it will be critical to dissect the role of multiple adaptors supporting HIV-1 replication in primary T cell lines instead of using non-T cell lines such as A549, HeLa, and CHME-5. Despite these limitations, our work provides testable hypotheses regarding HIV-1 with the dynein transport machinery and HIV-1 motility in cells.

Our work on HIV-1 reconstitution with dynein expands our understanding of HIV-1 trafficking and is consistent with the initial studies that linked BicD2 to HIV-1 infection (*12*, *13*). These initial studies showed that knockout or knockdown of BicD2 led to a reduction in infectivity for HIV-1. Both of these publications used cosedimentation to observe BicD2 cosediments with CA or CA-NC (nucleocapsid) assemblies in cell lysates (*12*, *13*) or CA assemblies in vitro (*12*). The work from the Aiken group showed that purified BicD2 truncations bound to CA assemblies in vitro, suggesting that multiple regions of BicD2 interact with CA. At this point, our work would suggest that these subregions within BicD2 do not play a role in motility, given that (i) BicD2^FL^ is not “opened up” by HIV-1 cores or CA assemblies to achieve dynein motility in our TIRF assay and (ii) the N-terminal coiled coils of BicD2 bind to dynein-dynactin for motility. Further work is needed to understand the relationship between HIV-1 capsid binding to BicD2 and whether this is important to infection and motility.

Building on these publications, our data suggest that BicD2 can be a cargo adaptor that supports HIV-1 motility. However, our model proposes that BicD2-mediated trafficking of HIV-1 occurs via HIV-1 binding to dynein directly and then moving with BicD2-cargoes in cells. Our model of trafficking proposes that HIV-1 will opportunistically use any active dynein when infecting different cell types and also highlights that knocking down adaptors in different cell types may have different effects. For instance, knockdown of Hook3 in A549 cells had a 36% decrease in infectivity, whereas knockdown of Hook3 in HeLa TZM-bl did not affect HIV-1 infection (Fig. 2F and fig. S3, G and H). Further research is needed to characterize the landscape of dynein motility across different cell types to determine which cargoes HIV-1 can exploit for retrograde transport. In addition, further investigation is required to explore the potential “direct binding of cargo adaptors” to other components of the HIV-1 core beyond its capsid.

To our knowledge, our reconstitution of HIV-1 trafficking via dynein represents the first example of viral trafficking using reconstituted components. Previous work on viral trafficking in vitro used lysate-based characterizations of motility, which helped to show directionality and motile behaviors (*58*–*60*). Lysate-based analyses, such as the one used for vaccinia virus (*60*), impose limitations on the ability to directly probe and isolate specific components of viral proteins or host cell factors necessary for motility. As highlighted in this work, our reconstituted system enabled us to perform dropout experiments with the dynein motor machinery in addition to mutating both HIV-1 capsid and dynein motor proteins.

Previous studies implicated direct binding of viral subunits to dynein motor proteins (*61*, *62*), such as the adenovirus hexon subunit binding to dynein (*63*). However, prior work did not establish the implication of direct viral binding to motor proteins. Here, our system allowed us to visualize HIV-1 exploiting multiple dynein cargo adaptors for motility to demonstrate that HIV-1 will opportunistically use any adaptors that enable dynein motility. The paradigm of viral hitchhiking on active motors may help place past and future work on viral trafficking within specific categories of opportunistic trafficking (e.g., HIV-1) versus directed trafficking, where a virus binds to a cargo adaptor directly to co-opt motor protein activity.

Beyond our work showing that HIV-1 binds to dynein directly, we leveraged protein engineering of bacterial encapsulins to design icosahedral protein cages to probe the effect of multiple dynein motors bound to a capsid-like shell (Fig. 5, E and F). Given that dynein trafficking in cells and in vitro is ~1 to 2 μm/s, we were surprised to see such a slow velocity for HIV-1 cores or capsids (~100 nm/s). The engineered encapsulin system allowed us to show that increasing the number of active dyneins on an encapsulin shell led to marked loss in velocities (Fig. 5E). From these data, we propose that a rigid cargo such as encapsulins or HIV-1 capsids affects dynein teams differently from fluid membrane–based cargoes such as vaccinia virus. We believe that fluid membrane cargoes allow for paused motors to be avoided by the dynein teams, whereas paused motors on rigid capsids lead to the pausing of the whole cargo-dynein team. Future work is needed to probe this model and to understand how this type of effect relates to kinesin-driven motility.

More broadly, our work shows that viruses such as HIV-1 can bypass interaction with a cargo adaptor by directly binding to motor proteins. Thus, direct motor protein hijacking may represent an important mode of trafficking for other viruses that use dynein and kinesin motor proteins.

## MATERIALS AND METHODS

### Protein expression, purification, and labeling

#### Dynein and dynactin purification

Bovine dynactin from cow brain and recombinant human cytoplasmic dynein expressed in insect cells were purified as described before (*64*). Human cytoplasmic dynein tail (DHC, 1 to 1450, with accessory proteins DYNC1I2, DYNC1LI2, DYNLT1, DYNLL1, and DYNLRB1), human cytoplasmic dynein motor domain (DHC, 1451 to 4500), and dynein-ΔDIC137 were all recombinantly expressed in insect cells. In summary, the baculovirus was prepared for dynein tail segments (pACEBac1 donor vector), dynein motor domain (pFastBac donor vector), and dynein-ΔDIC137 [DHC full length, with accessory proteins DYNC1I2 (138 to 640), DYNC1LI2, DYNLT1, DYNLL1, and DYNLRB1] in SF9 cells using standard methods. The proteins were expressed in SF9 or High Five cells (Thermo Fisher Scientific, BTI-TN-5B1-4) using passage one of viruses. Lysis buffer includes 50 mM Hepes (pH 7.4), 100 mM NaCl, 10% glycerol, 0.5 mM EGTA, 1 mM dithiothreitol (DTT), 0.1 mM ATP-Mg, 1 U of benzonase, SIGMAFAST protease inhibitor tablets + 0.5 mM Pefabloc. The proteins were bound to IgG (immunoglobulin G) Sepharose 6 Fast Flow (GE Healthcare) via the ZZ tag on the heavy chain. After washing with lysis buffer and DynBac TEV buffer [50 mM tris-HCl (pH 8), 2 mM Mg acetate, 1 mM EGTA, 250 mM K acetate, 10% glycerol, 1 mM DTT, and 0.1 mM ATP-Mg], proteins were eluted by treating the beads with at home-purified TEV protease in DynBac TEV buffer. The eluent was subjected to size exclusion chromatography using GF150 buffer [25 mM Hepes (pH 7.4), 150 mM KCl, 0.5 mM EGTA, and 1 mM DTT]. The collected fractions were concentrated and stored in a GF150 buffer including 10% glycerol at −80°C. SDS–polyacrylamide gel electrophoresis (PAGE) followed by Coomassie or silver staining was used to evaluate the purity, size, and the presence of the related polypeptide chains for each construct.

To analyze microtubule recruitment, the SNAP tag at the N terminus of the heavy chain in each construct was used for fluorescence labeling by SNAP-Cell TMR-Star or SNAP-Surface Alexa Fluor 647. The extra dye was removed by size exclusion chromatography. The efficiency of labeling was determined by NanoDrop and based on the protein concentration.

#### Full-length and truncated BicD2 and BicDR1

8×His-zz-TEV-mBICD2 (full-length or truncated 1 to 560) was codon optimized for expression in SF9 insect cells and synthesized by GenScript. The genes were subcloned into pFastBac using NEBuilder HiFi DNA assembly [New England Biolabs (NEB), E2621S]. zz-TEV-Halo-hBicD2^FL^-6×His in donor vector pKL for expression in insect cells and zz-TEV-Halo-hBicD2(1 to 598) in pET28a vector for expression in *Escherichia coli* were received from DeSantis Lab.

Baculoviruses were prepared from pFastBac or pKL (for Halo-BicD2^FL^) donor vectors using standard methods. SF9 cells at a density of 2 × 10^6^/ml were infected with passage 2 of virus at 1:100 ratio and harvested 48 hours later. Each gram of cell pellet was resuspended in 8 ml of buffer A [30 mM Hepes-KOH (pH 7.4), 150 mM KCl, 10% glycerol, 1 mM EGTA, and 10 mM β-Mercaptoethanol (BME)] in the presence of 0.1 mM phenylmethylsulfonyl fluoride (PMSF), SIGMAFAST protease inhibitor, and 25 U of benzonase. The cells were lysed, and the lysate was cleared as described for dynein. The clear lysate was incubated with washed IgG Sepharose Fast Flow beads (Cytiva) at 4°C for >2 hours to be purified via their ZZ tag. The BicD2 bound beads were washed with 100 ml of buffer A, including 0.02% NP-40, followed by 100 ml of wash with TEV buffer [10 mM tris (pH 8), 150 mM KCl, 1 mM EGTA, 1 mM DTT, and 10% glycerol]. The beads were resuspended in 0.5 to 1 ml of TEV buffer and incubated with in-home purified TEV protease (100 μg/ml) overnight. The eluent was subjected to further purification with size exclusion chromatography using Superose 6 300/10 in the presence of GF150 and lastly stored in a low-salt buffer [25 mM Hepes (pH 7.4), 50 mM KCl, 0.5 mM EGTA, and 5% glycerol].

The Halo-hBicD2 N-terminal domain (1 to 598) in the pET28a vector was expressed in *E. coli* using HyperBroth medium induced with 0.5 mM isopropyl-β-d-thiogalactopyranoside (IPTG). Cells were suspended and sonicated in lysis buffer [30 mM Hepes (pH 7.4), 100 mM NaCl, 2 mM Mg acetate, 0.5 mM EGTA, and 10% glycerol] in the presence of lysozyme (1 mg/ml), 1 mM DTT, and SIGMAFAST protease inhibitor. The lysate was cleared by a 45-min centrifugation at 100,000*g* at 4°C. IgG beads washed and equilibrated with the lysis buffer were added to the lysate. After a 2-hour incubation, the beads were collected and washed with lysis buffer + 1 mM DTT, followed by 100 ml of wash with TEV buffer [50 mM tris-HCl (pH 8), 2 mM Mg acetate, 1 mM EGTA, 250 mM K acetate, 10% glycerol, and 1 mM DTT]. The beads were suspended in 0.5 to 1 ml of TEV buffer and incubated with TEV protease at 100 μg/ml at 4°C overnight. The eluted protein was further purified by size exclusion chromatography using GF150. The fractions of Halo-hBicD2 (1 to 598) were combined, concentrated, and rebuffered into GF150, 1 mM DTT, and 10% glycerol. Halo-BicD2 (full-length or truncated) was labeled by HaloTag Alexa Fluor 660 ligand (Promega) by mixing protein solution with the dye at 1:2 molar ratio for 30 to 60 min at 4°C. Excess dye was removed by size exclusion chromatography.

8×His-ZZ-TEV-SNAP-mBicDR1^FL^ in pOmnibac vector was received from Addgene (catalog nos. 111859 and 111858). N-terminal truncated BicDR1 (1 to 400) was prepared from the full-length BicDR1 in the same vector by NEBuilder HiFi DNA Assembly. Baculoviruses were prepared using SF9 insect cells as described before. Passage 1 of viruses was used to express the proteins in High Five cells (Thermo Fisher Scientific). The cells harvested 48 hours postinoculation were lysed in 50 mM tris-HCl (pH 8), 150 mM NaCl, 10% glycerol, 1 mM EGTA, and 4 mM DTT supplemented with 0.2% NP-40 and benzonase in presence of protease inhibitor (SIGMAFAST and 0.1 mM PMSF). Clear lysates were incubated with IgG Sepharose Fast Flow beads (Cytiva) as described for BicD2 purification. Proteins were eluted from IgG beads using TEV protease (at 100 μg/ml) in TEV buffer [25 mM tris-HCl (pH 8), 50 mM NaCl, and 10% glycerol] after incubation at 16°C for 1 to 2 hours. For fluorescence labeling, eluated proteins from IgG beads were centrifuged at 17,000*g* for 45 min, and 500 μl of each protein was incubated with SNAP-Cell TMR-Star 555 in 1:3 molar ratio at 4°C for 2 hours. The labeled and unlabeled protein samples were further purified by size exclusion chromatography using Superose 6 300/10 equilibrated with 50 mM tris-HCl (pH 8.0), 50 mM NaCl, and 1 mM EGTA.

#### SpyCatcher BicDR1

The ZZ-BicDR1^FL^-SpyCatcher and ZZ-SNAP-BicDR1^FL^-SpyCatcher constructs in pFastBac donor vector were made by Gibson assembly using NEBuilder HiFi DNA Assembly. The baculoviruses were prepared using SF9 cells. High Five cells (Thermo Fisher Scientific) at 2 × 10^6^/ml were infected with passage 1 of virus. The pellet of the infected cells was collected after 52 hours and lysed in lysis buffer [50 mM tris (pH 8), 150 mM NaCl, 10% glycerol, 1 mM EGTA, 4 mM DTT, SIGMAFAST protease inhibitor, 0.1 mM PMSF, 10 U of benzonase, and 0.2% NP-40]. Clear lysates were incubated with IgG Sepharose 6 Fast Flow (GE Healthcare) for 4 hours at 4°C. The beads were collected and washed with 100 ml of lysis buffer (with 0.02% NP-40), then 100 ml of high-salt lysis buffer (250 mM NaCl), and lastly 50 ml of low-salt TEV buffer [50 mM tris-HCl (pH 8), 50 mM NaCl, and 10% glycerol]. Eventually, the beads were incubated with TEV protease in the TEV buffer for 2 hours at 16°C.

In the case of SNAP-BicDR1^FL^-SpyCatcher, the TEV-cut eluent protein was treated with SNAP-Surface Alexa Fluor 647. The proteins were further purified by Superose 6 Increase 10/300 GL column (Cytiva) using TEV buffer for equilibration. The fractions collected, concentrated, and stored in the same buffer as for gel filtration with 0.5 mM DTT and 10% glycerol.

#### Hook3

Human Hook3 (1 to 552) with ZZ-TEV-Halo tag at its N terminus was received as a gift from Reck-Peterson Lab (University of California San Diego) in pET28a vector. Proteins were expressed in BL21(DE3) *E*. *coli* strain in autoinduction medium (Terrific Broth Base including Trace elements, Formedium) at 25°C. The cell pellet were lysed at 50 mM Hepes (pH 7.4), 150 mM NaCl, 10% glycerol, 1 mM EGTA, 20 mM BME, SIGMAFAST protease inhibitor, 0.1 mM PMSF, 10 U of benzonase, and 5 mg of lysozyme. Cleared lysate was incubated with washed IgG Sepharose 6 Fast Flow (GE Healthcare) for 3 hours at 4°C. The beads were collected and washed in tandem with lysis buffer, high-salt lysis buffer (300 mM NaCl), lysis buffer, and eventually low-salt TEV buffer [25 mM tris-HCl (pH 8), 50 mM NaCl, 5% glycerol, 0.5 mM EGTA, and 0.5 mM EGTA]. The proteins released from the IgG beads after overnight incubation with TEV protease in the low-salt TEV buffer. For fluorescently labeled Hook3, 300 μl of eluant (at 1.5 mg/ml) was treated with 6.5 μl of 1 mM HaloTag Alexa Fluor 488 ligand (Promega, PAG1001). Both labeled and unlabeled proteins were subject to size exclusion chromatography for further purification using Superose 6 Increase 10/300 GL column (Cytiva) equilibrated in 50 mM tris-HCl (pH 8) and 50 mM NaCl. The fractions collected, concentrated, and stored at −80 in 50 mM tris-HCl (pH 8) and 50 mM NaCl with 6% glycerol.

#### Ninein-like

NINL (1 to 702) containing an N-terminal HaloTag was expressed in BL-21(DE3) cells (NEB), which were then grown until the optical density at 600 nm (OD_600_) of 0.4 to 0.6 and induced with 0.1 mM IPTG for 16 hours at 16°C. Frozen cell pellets from a 2-liter culture were resuspended in 60 ml of activator lysis buffer [30 mM Hepes (pH 7.4), 50 mM potassium acetate, 2 mM magnesium acetate, 1 mM EGTA, 1 mM DTT, 0.5 mM Pefabloc, and 10% (v/v) glycerol] supplemented with 1 cOmplete EDTA-free protease inhibitor cocktail tablet (Roche) per 50 ml and lysozyme (1 mg/ml). The resuspension was incubated on ice for 30 min and lysed by sonication. The lysate was clarified by centrifuging at 66,000*g* for 30 min in Type 70 Ti rotor (Beckman). The clarified supernatant was incubated with 2 ml of IgG Sepharose 6 Fast Flow beads (Cytiva) for 2 hours on a roller. The beads were transferred to a gravity flow column, washed with 100 ml of activator lysis buffer supplemented with 150 mM potassium acetate, followed by 50 ml of cleavage buffer [50 mM tris–HCl (pH 8.0), 150 mM potassium acetate, 2 mM magnesium acetate, 1 mM EGTA, 1 mM DTT, 0.5 mM Pefabloc, and 10% (v/v) glycerol]. The beads were then resuspended and incubated in 15 ml of cleavage buffer supplemented with TEV protease (0.2 mg/ml) overnight on a roller. The supernatant containing cleaved proteins was concentrated using a 30,000 molecular weight (MW) cutoff (MWCO) concentrator (EMD Millipore) to 1 ml, filtered by centrifuging with Ultrafree-MC VV filter (EMD Millipore) in a tabletop centrifuge, diluted to 2 ml in buffer A [30 mM Hepes (pH 7.4), 50 mM potassium acetate, 2 mM magnesium acetate, 1 mM EGTA, 10% (v/v) glycerol, and 1 mM DTT], and injected onto a MonoQ 5/50 GL column (Cytiva) at 1 ml/min. The column was prewashed with 10 column volume (CV) of buffer A, 10 CV of buffer B [30 mM Hepes (pH 7.4), 1 M potassium acetate, 2 mM magnesium acetate, 1 mM EGTA, 10% (v/v) glycerol, and 1 mM DTT] and again with 10 CV of buffer A at 1 ml/min. To elute, a linear gradient was run over 26 CV from 0 to 100% buffer B. The peak fractions containing Halo-tagged activating adaptors were collected and concentrated using a 30,000 MWCO concentrator (EMD Millipore) to 0.2 ml, diluted to 0.5 ml in GF150 buffer [25 mM Hepes (pH 7.4), 150 mM potassium chloride, 1 mM magnesium chloride, and 1 mM DTT], and further purified via size exclusion chromatography on a Superose 6 Increase 10/300 GL column (Cytiva) with GF150 buffer at 0.5 ml/min. The peak fractions were collected, and buffer was exchanged into a GF150 buffer supplemented with 10% glycerol, concentrated to 0.2 to 1 mg/ml using a 30,000 MWCO concentrator (EMD Millipore), and flash frozen in liquid nitrogen.

For fluorescent labeling of the HaloTag, the purified protein was incubated with 50 μM Halo–Alexa Fluor 488 (Promega) for 10 min at room temperature. Free dye was removed using a micro bio-spin chromatography column (Bio-Rad) equilibrated in GF150 buffer supplemented with 10% glycerol. The labeled protein was flash frozen in liquid nitrogen.

#### *Dynein light chains* (*LC8 and TCTex1*)

DYNLL1 dynein light chain [glutathione *S*-transferase (GST)–PreScission Site–LC8] was obtained in the pHH0103 vector backbone from Addgene (catalog no. 110006) and transfected into the BL21(DE3) *E. coli* strain for bacterial expression. Protein expression was induced with 0.5 mM IPTG at 21°C for 16 hours. The cell pellet was lysed in LC8 lysis buffer [containing 3% glycerol, 100 mM NaCl, 50 mM Hepes (NaOH) (pH 7.4), 0.5 mM EGTA, and 1 mM DTT, along with lysozyme, protease inhibitor, and benzonase]. The clarified lysate was then applied to a GSTrap HP Column, and the bound GST-LC8 was eluted using a gradient of reduced glutathione (up to 20 mM) in the lysis buffer.

For tag-free LC8, 1 to 2 ml of glutathione resin was mixed with 40 ml of the clear lysate and allowed to bind for 2 hours at 4°C on a roller. After binding, the mixture was added to a disposable 10-ml column and washed with 100 ml of 1× lysis buffer. The resin was then resuspended in ~1 ml of fresh lysis buffer, and 20 μl of PreScission protease (GenScript, catalog no. Z02799-250) was added. The column was sealed and incubated on a roller at 4°C overnight. The eluent resulting from protease activity was then purified using a Superdex 200 Increase 10/300 GL column (catalog no. 28-9909-44) equilibrated with GF75 buffer. The size, purity, and concentration of the LC8 protein were confirmed by SDS-PAGE analysis.

Recombinant human TCTEX-1 protein (DYLT1 full length) was obtained as a purified sample from Abcam (catalog no. ab106872). The protein was expressed in an *E. coli* system, purified to over 95% purity using an N-terminal His tag, and stored in a buffer containing 50 mM tris-HCl (pH 8.0), 100 mM NaCl, 30% glycerol, and 1 mM DTT at −80°C.

### Preparation of fluorescent HIV-1 cores and capsids

#### HIV-1 core purification

GFP-labeled HIV-1 cores were purified from HIV-1 particles generated from the Env-defective proviral clone (R9ΔE) and a plasmid expressing the GFP-Vpr fusion protein (*6*). Viruses were produced by transfection of 4 × 10^6^ 293T cells seeded in 100-mm dishes with 10 mg of R9∆E plasmid and 1 mg of GFP-Vpr plasmid. HIV-1 cores were purified from virions concentrated from 30 ml of culture supernatant as described previously (*65*). Aliquots of purified cores were snap frozen and stored at −80°C. Samples were thawed on ice before use in motility assays.

#### Recombinant CA purification

HIV-1 CA (A14C/E45C), CA (A92E/A204C), and CA (A92E/A204C/R18G) in pET21a plasmid and CA (K156C) in pET11a plasmids were expressed and purified from the *E. coli* BL21(DE3) strain. The transformed bacteria grow to OD_600_ ≈ 2 in HyperBroth medium before induction with 0.5 mM IPTG. The cells were collected after overnight shaking at 23°C. Purification steps were followed as explained before (*66*). Lysis buffer included 50 mM tris-HCl at pH 8, 50 mM NaCl, lysozyme (2 mg/50 ml), benzonase (1.5 μl/50 ml), 10 mM TCEP [tris(2-carboxyethyl)phosphine], 10 mM DTT, and SIGMAFAST protease inhibitors. Solid ammonium sulfate (Sigma-Aldrich, #A4418) was added slowly to clear lysate to reach 27% saturated ammonium sulfate. After 30 min of stirring, the pellet of precipitated proteins was collected at 40,000*g* and then resuspended in 25 mM Hepes (pH 7.2) and 15 mM TCEP. After overnight dialysis in the same buffer, the solution was centrifuged at 70,000*g*, and the supernatant of solubilized proteins was injected onto a HiTrap SP HP cation exchange column (Cytiva). The loaded column was washed with no-salt buffer, and the CA was eluted from the column gradually with over 15 column volumes of 25 mM Hepes and 1000 mM NaCl. The eluted proteins were dialyzed in the low-salt buffer overnight. The final protein sample was concentrated to 10 to 20 mg/ml and evaluated by SDS-PAGE.

#### CA assembly and fluorescence labeling

Cross-linked CA (A14C/E45C) tube-shaped constructs were formed by three tandem overnight dialysis of CA (5 to 10 mg/ml) as described before (*67*): (i) assembling buffer: 25 mM tris (pH 8.0), 1 M NaCl, and 30 mM DTT; (ii) the same as assembling buffer omitting DTT; and (iii) low-salt buffer: 25 mM tris (pH 8.0) and 50 mM NaCl. HIV-1 CA cones were formed by dialysis of CA (A92E/A204C) in the assembly buffer [5 mM IP_6_, 30 mM MES (pH 6), 30 mM NaCl, and 30 mM BME]. The disulfide bonds at the interface of the hexameric units were formed by letting another overnight dialysis in the absence of BME. Eventually, the formed cones were dialyzed in 30 mM MES (pH 6) and 30 mM NaCl to remove the extra IP_6_. Capped tube-shaped CA (A92E/A204C) and CA (A92E/A204C/R18G) were formed in the same manner as HIV-1 CA cones, and only IP_6_ was replaced by 1 M NaCl.

The method by Lau *et al.* (*68*) was followed to create fluorescent HIV-1 capsid. In summary, CA (K158C) was assembled into tubes in the presence of a labeling buffer [50 mM tris (pH 7.4), 2.5 M NaCl, and 0.3 mM TCEP]. Then, the tube was incubated for 2 min with a 2× excess molar ratio of fluorescent dye (DyLight 488 Maleimide) at room temperature in the dark. Extra dye was quenched with 25 mM βME (final), and the labeled CA tubes were pelleted and resuspended in fresh labeling buffer. After several steps of pelleting and resuspending to remove the unreacted dye, the tubes were disassembled in the no-salt labeling buffer. Fluorescently labeled CA (K158C) was aliquoted and stored at −80°C.

To label the HIV-1 CA tubes or cores, labeled CA (K158C) was mixed with unlabeled CA at 1:40 or 1:80 molar ratio before assembling. After the final dialysis, the tubes or cones were centrifuged at 17,000*g* for 10 min to remove the free CA (K158C). The pellet was resuspended in 50 mM Hepes (pH 7.2) and 150 mM NaCl. Labeled CA tubes were stored at 4°C in the dark. For CA cones, the resuspended cones were centrifuged at 4000*g* for 5 min. The supernatant included labeled “soluble” cones and was stored at 4°C. The integrity of tubes and cones was evaluated by negative staining. The concentration of tube and cone (as assembled particles) was calculated on the basis of the final CA contraction and the length of tubes/cones as measured by ImageJ (*69*).

### TIRF imaging

#### HIV-1 microtubule recruitment and motility assay

Single-molecule assay and microtubule-binding assays were performed in flow chambers assembled as described previously (*70*). Biotin–polyethylene glycol (PEG)–functionalized coverslips were made in the laboratory following described procedures (*71*) with some modifications. In summary, coverslips (1.5 refractive index) were sonicated in 200-proof ethanol at 40°C for 10 min. After washing with Milli-Q water, the coverslips were sonicated in 200 mM KOH at 40°C for 20 min, followed by washing with Milli-Q and another round of sonication with ethanol (Thermo Fisher Scientific, lot A995-4, high-performance liquid chromatography grade). The incubation solution was prepared by mixing methanol (Thermo Fisher Scientific, lot A452-4), acetic acid (Thermo Fisher Scientific, lot A35-500), and aminosilane (lot A01W32000610) in 20:5:1 volumetric ratio. Coverslips from the last step were incubated overnight in the incubation solution in the dark and dry place. Then, coverslips were washed with water and ethanol and dried out before treating with PEG-biotin solution. PEG solution included mPEG–succinimidyl valerate (0.25 g/ml) (MW, 2000; Lysanbio), biotin-PEG-Succinimidyl Valerate (0.02 g/ml) (MW, 5000; Lysanbio) in 0.84% (w/v) sodium bicarbonate solution. PEG solution (60 μl) was added to a clean ethanol-washed slide, and one of the above coverslips was laid down on that 60 μl of solution and incubated for 2 to 3 hours in the dark. Then, the coverslips and slides were washed with Milli-Q water and kept in the pair in a vacuum for future use. Biotin-PEG–functionalized coverslips were stable for at least 1.5 months after preparation.

Microtubules with ∼5% biotin-tubulin (Cytoskeleton Inc.) and ∼5% Alexa Fluor 488–labeled or Alexa Fluor 647–labeled fluorescent tubulin (Cytoskeleton Inc.) were prepared as described (*72*) and stabilized with 20 μM final taxol (Cytoskeleton Inc.). The flow chamber was incubated sequentially with streptavidin (1 mg/ml) and then with diluted, taxol-stabilized microtubules (final tubulin at 0.025 mg/ml) with a 5-min incubation time. After each step, the chamber was washed twice with the BRB80 buffer [80 mM K Pipes, 1 mM EGTA, and 2 mM MgCl_2_ (pH 6.8), supplemented with 20 μM taxol].

Single-molecule TIRF microscopy was performed using Olympus inverted microscope at ×60 magnification with no further magnifier. The microscope was equipped with Hamamatsu charge-coupled device (CCD) digital camera at 16-μm pixel size. Olympus cellTIRF-4Line system provided four laser channels with independent beam paths and depth. Semrock single-band BrightLine filters were used for the excitation path filter, and 405/488/561/647-nm Laser Quad Dichromic Cube (Chroma, TRF89902-ET) was the integral emission filter. Olympus CellSens Dimension software controlled the image and movie acquisition, and the z-focus was controlled manually. In all experiments, laser power was set to 15 to 25% for all laser beams, which measured 3.5 to 9.2 mW for 488-nm laser, 2.4 to 5.8 mW for 561-nm laser, and 4.2 to 12.9 mW for 647-nm laser.

All imaging was acquired in the dynein-lysis buffer (DLB) [25 mM Hepes (pH 7.4), 2 mM Mg acetate, 10% glycerol, and 1 mM EGTA] and 20 μM taxol. Mg ATP was kept at 1 mM unless otherwise noted. For consistency, no reducing agent was used in the imaging solution. The oxygen scavenging system was based on the gloxy combination of glucose oxidase and catalase enzymes (*73*).

The complexes were assembled by mixing HIV-1 cores (GFP-Vpr) or fluorescently labeled HIV-1 cone-shaped/tube-shaped capsids with dynein for 10 min on ice, and then dynactin and activating adapter were added and incubated for an additional 10 min. Core:activator:dynactin:dynein were mixed in 1:200:500:1000 ratio. The final salt concentration in all experiments was kept at 30 to 35 mM.

HIV-1 (core or capsid) and microtubules were imaged with 500-ms intervals and 50-ms exposure for each fluorescent channel. All single-molecule assays were recorded for 4 min, unless otherwise mentioned. Velocities, bindings, and processivity were calculated from kymographs using Fiji/ImageJ (*74*) as described before (*75*). The total number of bindings per microtubule (at each field of view for each replicate of the experiment) was divided per imaging time (in minutes), length of the microtubule (in micrometers), and the concentration of HIV-1 core or capsid (full complex, in nanomolars) to calculate the binding rate without any further normalization. Binding and velocity data were collected from at least 20 (on average, 25) microtubules. For statistical calculations, we only used data from the very first 4-min movie of each experiment. This approach helped minimize variability in the system, which could arise because of ATP depletion or increased nonspecific binding over time. For statistical analysis, we performed biological replicates by repeating the experiment with a fresh imagining mixture and collecting a new first 4-min movie from each subsequent experiment. We repeated the experiment at least twice, typically three to four times, to obtain a total of at least 20 individual, fully extended microtubules for analysis. Processivity percentage refers to measuring the proportion of HIV-1 particles that exhibit processive motion (continuous, directed movement along microtubules) relative to the total number of HIV-1 particles that bind to microtubules (including diffusive, unmoved, etc.).

#### Nup358^min-zip^ microtubule recruitment and motility assay

Nup358^min-zip^ construct [GST–PreScission Site–hNup358(2148 to 2240)–GCN4–SnapTag] for bacterial expression was provided by Solmaz laboratory (Binghamton University) in pGEX-6p1 plasmid. It was expressed and purified following the previously described protocol (*16*). Briefly, Nup358^min-zip^ was expressed in codon plus *E*. *coli* (BL21 bearing pRare plasmid under arabinose operon) using autoinduction medium (Terrific Broth Base including Trace elements, Formedium) at 20°C. The cell pellet from 5 liters of culture was lysed in lysis buffer [10% glycerol, 150 mM NaCl, 50 mM Hepes (NaOH) (pH 7.4), 1 mM EGTA, 1 mM DTT, and protease inhibitor]. The clarified lysate was incubated with glutathione resin for 2 hours at 4°C. Then, the resin was collected, washed, and incubated with 10 μl of PreScission protease (GenScript, catalog no. Z02799-250) overnight at 4°C. The eluted Nup358^min-zip^ was labeled by Snap-Surface 488 (NEB, #S9124S) and further purified by size exclusion chromatography using a Superdex200 column equilibrated with GF150 buffer to separate it from the unreacted dye.

The motility assay of Nup358^min-zip^ in presence of dynein, dynactin, and full-length hBicD2 was performed according to the method described previously (*16*). BicD2 and labeled Nup358^min-zip^ were combined in a 1:1 molar ratio and incubated on ice for 15 min. Next, BicD2-Nup358^min-zip^ complex was mixed with dynein-dynactin to achieve a final molar ratio of 1:1:2:2, respectively. The mixture was incubated on ice for 30 min, before being diluted to a final concentration of 10 nM dynein in the imaging solution (the same solution used for the HIV-1 assays). Recruitment of Nup358^min-zip^ labeled at 488 nm to microtubules (labeled with Alexa Fluor 647) and its trafficking in the presence of dynein, dynactin, and full-length BicD2, or in the absence of either dynein or BicD2, was imaged and monitored exactly as described in the previous section for the HIV-1 microtubule recruitment and motility assay.

#### Competition experiments

To figure out the binding site(s) of dynein on HIV-1 capsid, motility assay was performed in presence of IP_6_ (phytic acid sodium salt hydrate, Sigma-Aldrich, #P8810) and PF74 (PF-3450074, MedKoo Biosciences, #555391). IP_6_ is known to coordinate rings of electropositive charge in the central pores of HIV-1 hexamers that interact with K25 and R18 rings at the pore. PF74 is a small molecule that binds to FG-binding pocket. Cone-shaped HIV-1 capsid was assembled with IP_6_ and fluorescently labeled as described in “CA assembly and fluorescence labeling” sections. IP_6_ eventually was removed by dialysis, pelleting, and resuspension. The motility assay was set up with dynein (10 nM), dynactin, and BicDR1 as described before. IP_6_ (made at 500 mM stock solution and pH adjusted to 6) was added as the last component to the imaging mixture at 10 or 100 μM final concentration and incubated on ice for 10 min before recording HIV-1 capsid motility. PF-74 stock solution was made freshly in dimethyl sulfoxide. PF74 was also added at a final concentration of 1 or 10 μM to the imaging solution as the last component and incubated on ice for 10 min before imaging. Velocities, bindings, and processivity were calculated and compared in presence of each putative competitor. PF74 has a *K*_d_ of 0.6 μM for binding to the HIV-1 capsid. In the competition experiment we conducted, the total CA concentration was ~75 nM in the imaging solution. Given these conditions, PF74 would occupy around 61% of the binding sites on the capsid at a concentration of 1 μM and about 95% at 10 μM, and from that 10 μM PF74, only ~0.07 nM is taken to saturate 95% of the binding sites on the capsid. Thus, nearly all binding sites on the capsid should be saturated at a PF74 concentration of 10 μM.

Two separate mutations of CA, N57D and G89V, were introduced into the original CA A92E/A204C in the pET21a^+^ plasmid using the QuikChange Lightning Site-Directed Mutagenesis Kit (catalog no. 210518). WT CA was synthesized and subcloned into the pET21a ^+^ plasmid by GenScript. Both WT and the mutant CAs were expressed and purified in *E. coli* as previously described. Cone-shaped HIV-1 capsids were assembled with IP_6_ and fluorescently labeled as outlined in “CA assembly and fluorescence labeling” sections. For the N57D/A92E/A204C and G89V/A92E/A204C CA mutants, IP_6_ was removed by dialysis, while for WT CA, IP_6_ was maintained at a final concentration of 40 μM until the final step of TIRF imaging.

### Encapsulin viral-like model system

#### Engineered encapsulin: Design and purification

A synthetic two-gene operon for encapsulin-SpyTag expression was designed containing mNeonGreen, N-terminally fused to the native IMEF cargo protein from *Quasibacillus thermotolerans* (UniProt ID: A0A0F5HNH9), separated by a GGSGGS linker, and the *Q. thermotolerans* encapsulin (UniProt ID: A0A0F5HPP7) with a C-terminal SpyTag (SAHIVMVDAYKPTK). The synthetic operon was codon optimized for *E. coli* overexpression and ordered as a gBlock gene fragment from Integrated DNA Technologies. The gBlock was designed to contain a 20 base pairs overlap with the multiple cloning site 2 of the pETDuet-1 expression vector. The expression plasmid was assembled via Gibson assembly by mixing pETDuet-1 vector digested with Nde I and Pac I with the gBlock in addition to Gibson assembly mix (NEB, #E2611L) and incubating at 50°C for 15 min. Assembled plasmid was inserted into electrocompetent *E. coli* DH10B cells by transformation via electroporation. The plasmid was confirmed via Sanger sequencing (Eurofins).

For expression of the encapsulin-SpyTag protein, 500 ml of terrific broth in a 2-liter baffled flask containing ampicillin (100 μg/ml) was inoculated with a 10-ml overnight culture of *E*. *coli* BL21 (DE3) containing the encapsulin-SpyTag expression plasmid. The culture was grown with shaking at 200 rpm at 37°C until it reached an OD_600_ of 0.5. Protein expression was then induced by the addition of 0.2 mM IPTG, and the culture was then moved to 30°C and grown with shaking at 200 rpm for 22 hours. Cells were then harvested by centrifugation, and the cell pellet was immediately lysed by sonication in a lysis buffer consisting of 150 mM NaCl, 20 mM tris (pH 8.0), 1 mM MgCl_2_, 1 mM CaCl_2_, 1× SIGMAFAST EDTA-free protease inhibitor cocktail, lysozyme (0.5 mg/ml), and deoxyribonuclease I (10 μg/ml). Sonication was performed using a Model 120 Sonic Dismembrator (Thermo Fisher Scientific) at 48% amplitude for 5 min total (10-s on and 20-s off) on ice. Cell debris was removed from the lysate by centrifugation at 10,000*g* for 10 min at 4°C. The clarified supernatant was then incubated on ice with gentle rocking for 30 min. Solid PEG-8000 [10% (w/v) final concentration] and 0.6 M NaCl were added to the supernatant, followed by incubation with gentle rocking at 4°C for 45 min. The precipitate was pelleted by centrifugation at 8000*g* for 15 min at 4°C, resuspended in 5 ml of 150 mM NaCl and 20 mM tris (pH 8.0), and filtered with a 0.2-μm syringe filter. The filtered protein sample was then subjected to size exclusion chromatography on a HiPrep 16/60 Sephacryl S-500 size exclusion column (GE Healthcare) using a running buffer consisting of 150 mM NaCl and 20 mM tris (pH 8.0). Encapsulin-containing fractions were pooled, concentrated, and desalted into 20 mM tris (pH 8.0) using an Amicon Ultra-15 centrifugal filter (EMD Millipore) with a 100-kDa MWCO and further purified by ion exchange chromatography using a HiPrep DEAE FF 16/10 ion exchange column (GE Healthcare). After loading, the column was washed with 100 ml of 20 mM tris (pH 8.0), followed by a 200-ml linear gradient to 1 M NaCl and 20 mM tris (pH 8.0). Encapsulin-containing fractions were again concentrated and exchanged into 150 mM NaCl and 20 mM tris (pH 8.0) using an Amicon Ultra-15 centrifugal filter with a 100-kDa MWCO and subjected to a final round of size exclusion chromatography using a Superose 6 Increase 10/300 GL size exclusion column (GE Healthcare) with 150 mM NaCl and 20 mM tris (pH 8.0) as the running buffer. All chromatography steps were performed using an ÄKTA Pure FPLC system (GE Healthcare). Encapsulin-containing fractions were then pooled, concentrated to 3.3 mg/ml according to absorbance at 280 nm, and immediately drop frozen in 25 to 30 μl of aliquots in liquid nitrogen. The frozen protein was then stored at −80°C until needed.

#### Encapsulin-BicDR1: Motility data

Purified BicDR1^FL^-SpyCatcher was incubated with encapsulin-SpyTag. The incubation buffer includes 50 mM tris (pH 8.0), 50 mM NaCl, 1 mM EDTA, 9% glycerol, and 0.5 mM DTT at the final volume of 21.5 μl. The encapsulin-SpyTag concentrations were kept constant at 15.5 nM, while the concentration of BicDR1^FL^-SpyCatcher adjusted to keep the ratio of 1, 2.5, 10, 25, and 100 BicDR1 (as a dimer) per encapsulin. The solutions were incubated at 4°C for at least 24 hours. To evaluate the yield of the reaction and to remove the unreacted BicDR1, the complexes of encapsulin-BicDR1 (~9 to 25 MDa) were diluted and subjected to ultrafiltration using 1-MDa MWCO Vivaspin. The formation of encapsulin-SpyTag-BicDR1^FL^-SpyCatcher complexes was analyzed and further quantified by SDS-PAGE. We confirmed the formation of a new band related to encapsulin-SpyTag-BicDR1^FL^-SpyCatcher even at the lowest concentration of BicDR1. On the basis of the ultrafiltration and SDS-PAGE, all added BicDR1-SpyCatcher was incorporated into encapsulin-SpyTag.

TIRF experiments of encapsulin-SpyTag-BicDR1^FL^-SpyCatcher with Enc:BicDR1(dimer) ratios of 1:1, 1:10, and 1:100 were conducted using the same buffer condition as was used for HIV-1 (DLB buffer, with 30 mM salt, 1 mM ATP, 20 μM taxol, and gloxy). Dynein and dynactin were kept constant at the saturation concentration, while the concentration of encapsulin-SpyTag-BicDR1^FL^-SpyCatcher construct was adjusted according to the number of BicDR1 per encapsulin. For example, we calculated how much of the Enc:BicDR1 construct at each ratio needs to be added to keep the final total BicDR1 in the imaging solution at 0.5 nM. In this way, the molarity and overall ratio of dynein, dynactin, and BicDR1 remained exactly the same in all single-molecule assays, while the concentration of encapsulin itself (the monitored single particles) in the imaging solution was 0.5, 0.05, and 0.005 nM for 1:1, 1:10, and 1:100 Enc:BicDR1 experiments, respectively.

Taxol-stabilized microtubules (labeled with Alexa Fluor 647 and 5% biotin-tubulin) were immobilized on the PEG-biotin–treated coverslip as described before in this manuscript. Imagining solution including encapsulin-BicDR1 was introduced into the chamber with immobilized microtubules and immediately imaged by TIRF microscope. A 488-nm laser was used for the excitation in the encapsulin channel (mNeonGreen). All the movies were recorded for 4 min with 500-ms interval and 50-ms exposure for 488- and 647-nm excitation. The velocity and binding rate were calculated from the extracted kymographs using Fiji/ImageJ (*74*).

#### Standard curve creation by encapsulin-BicDR1 and quantification of dyneins per HIV-1 core

Encapsulin-SpyTag-SNAP-BicDR1^FL^-SpyCatcher complex was used to generate the standard curve to calculate the number of dyneins bound to the HIV-1 core. The standard curve is supposed to plot the fluorescent intensity per encapsulin when a different number of fluorescently labeled BicDR1 is bound to encapsulin. To do so, purified SNAP-BicDR1^FL^-SpyCatcher was labeled with SNAP-Surface Alexa Fluor 647 at almost 100% labeling yield. To generate encapsulin-SpyTag-SNAP^Alexa647^-BicDR1^FL^-SpyCatcher, the same incubation conditions, preparation, and evaluation steps as described for BicDR1^FL^-SpyCatcher were followed. Enc:SNAP^Alexa647^-BicDR1 at 1:1, 1:2.5, 1:10, 1:25, and 1:100 ratios was diluted at least 200 times in the imaging buffer (DLB with 33 mM salt, 1 mM ATP, and gloxy system) to make a layer of separated single particles on the untreated coverslip. Then, several images were immediately taken at 50-ms exposure from different spots on the coverslip at 647-nm excitation. Images were processed with Fiji/ImageJ software. A region of interest (ROI) was created and applied to all the images to measure the fluorescence intensities of single particles at the region away from the edge of the image. The ComDet (*76*) plugin installed in Fiji was used to detect spots and analyze colocalization. The ComDet parameters for colocalization analysis including distance between colocalized spots (4 pixels), approximate particle size (4 pixels), and intensity threshold (*2*) were applied, and large particles were segmented. The fluorescence intensity (in arbitrary units) at 647 nm were calculated and collected only from the particles colocalized with 488-nm fluorescence intensity (encapsulin) and then normalized by subtracting the background fluorescence intensity at 647 nm for each image. A histogram plot was generated from the normalized intensity data for each of Enc:SNAP^Alexa647^-BicDR1, and mean values were determined by fitting a Gaussian curve to histogram data. By plotting these mean values against the number of SNAP^Alexa647^-BicDR1 per encapsulin, the standard curve was formed.

WT dynein was also fluorescently labeled with SNAP-Surface Alexa Fluor 647 via the SNAP tag at the N terminus of DHC with 93% labeling efficiency. Then, dynein at 10 nM was incubated with 0.05 nM HIV-1 cores (GFP-Vpr) in the buffer similar to TIRF assay (DLB with 30 mM salt, 1 mM ATP, and gloxy system). The mixture was incubated on ice for 20 min and then diluted 20 times in the same incubation buffer before infusing the solution into a chamber of untreated coverslip. Imaging at 647 nm was performed following exactly the same conditions and microscope settings as used for encapsulin-SpyTag-SNAP^Alexa647^-BicDR1^FL^-SpyCatcher. Images were processed by applying the same ROI and ComDet parameters as used for encapsulin-SpyTag-SNAP^Alexa647^-BicDR1^FL^-SpyCatcher. Histogram graphs from the normalized fluorescence intensity at 647 nm for single particles colocalized with HIV-1 core (at 488-nm excitation) were generated, and the mean value of this fluorescence intensity was calculated from the fitted Gaussian curve to the histogram graph. The number of dyneins per HIV-1 core was calculated by using the trendline equation of encapsulin-SpyTag-Snap^Alexa647^-BicDR1^FL^-SpyCatcher standard curve and applying the labeling efficiency of dynein.

### In vitro cosedimentation assays

Cross-linked CA (A14C/E45C) was prepared as described in the previous section. Purified dynein, dynein tail domain, dynein motor domain, DLC TcTEx1, and DLC LC8 were centrifuged at 17,000*g* for 30 to 60 min at 4°C before copelleting. Each protein was added to CA tubes (equal to ~30 μM CA hexamer) in 40-μl final volume. DLB with 0.1 mM ATP and the final salt of 50 mM was used as an incubating buffer. The mixtures were incubated for 60 min at 4°C on the roller. Subsequently, 10 μl of aliquots were withdrawn and labeled as total. The remaining was centrifuges at 4°C for 7 min at 40,000*g*. The pellet resuspended in 30 μl of loading buffer + BME. Ten microliters of total, supernatant, and pellet samples were analyzed by SDS-PAGE.

### Electron microscopy

All negative stain imaging in this paper was performed using a Morgagni 268(D) transmission electron microscope S3 microscope (FEI Co.). The microscope is equipped with a tungsten filament operated at 100-kV high tension and a Gatan Orius SC200 CCD detector with a physical pixel size of 7.4 μm. Images well acquired at nominal magnification of ×22,000 (2.1 Å per pixel) at room temperature. The samples were fixed using conventional negative staining procedures with 0.075% uranyl formate on glow-discharged grids with Formvar carbon film (Electron Microscopy Sciences, FCF-400-Cu-50).

### Cell-based HIV-1 infection assays

#### Small interfering RNA knockdowns

Small interfering RNA (siRNA) targeting BicDR1 (Ambion siRNA #149588), Hook3 (Ambion siRNA #s228364), or negative control siRNA (negative control no. 1, Thermo Fisher Scientific, #4390843) was purchased from Thermo Fisher Scientific. siRNAs were transfected twice into cells 24 hours apart using Lipofectamine 3000 (Invitrogen) as per the manufacturer’s protocol. Briefly, 300 pmol of siRNA was added to OptiMEM medium containing 7.5 μl of Lipofectamine 3000 and incubated for 15 min at room temperature. Following incubation, the siRNA mixture was added dropwise to cells and allowed to incubate for 12 hours in medium without antibiotics before replacing medium. Cells were allowed to rest for 8 hours before the second transfection was performed using the same protocol. Following the conclusion of the second transfection, cells were collected for confirmation of knockdown via quantitative polymerase chain reaction (qPCR) or Western blot and subsequent experiments.

To confirm siRNA knockdown, Western blot or qPCR analysis was performed. For Western blot, siRNA-treated cells were lysed 12 hours following the second siRNA transfection. Western blot was performed using the following antibodies: mouse anti-Hook3 (sc-398924). For qPCR, RNA was isolated and purified from cells using the NucleoSpin RNA extraction kit (Macherey-Nagel). Following RNA isolation, cDNA conversion was performed using the GoScript Reverse Transcriptase Kit (Promega). qPCR was performed using separate sets of primers amplifying various regions of each gene. The primer sets were as follows: BICDR1 #1, 5′-GAG CTG GAG AGT GAT GTG AAG-3′ (forward) and 5′-CCT GCT GAG CTG ATC CAA TAG-3′ (reverse); BICDR1 #2, 5′-CAT CAA CCA ACC AGC ACA TTA TC-3′ (forward) and 5′-TCC TCT AAA GTA GCG CTG AGA-3′ (reverse); BICDR1 #3, 5′-CAA GTC ATC TGC AGG CTT TA-3′ (forward) and 5′-GTT TGG CTA GGC CAA GAA ATT AT-3′ (reverse); BICDR1 #4, 5′-CGA GCA CTT AGA GCA AGA GAA A-3′ (forward) and 5′-GTA GCT GCT TCA CAT CAC TCT C-3′ (reverse); BICDR1 #5, 5′-ACA TCC CTC CTG TCA GAG AT-3′ (forward) and 5′-CGA AGG TGT GAG CAC AGA TAG-3′ (reverse). Hook3 #1, 5′-GAT CAG TAG TGA GTG AGT GTT AAG T-3′ (forward) and 5′-AGG AGG AAG GAA GGA GAG ATA G-3′ (reverse); Hook3 #2, 5′-GTG GAG TGG ATC ATT GGG ATA AG-3′ (forward) and 5′-CTA TTG CTT TGG GAA CAT CTG TTT AG-3′ (reverse); Hook3 #3, 5′-AGG AGC AGC ACC AGA AAT AC-3′ (forward) and 5′-CTT CCA TCT CTC TCT GAG TCT TTG-3′ (reverse).

#### Virus production and infection analysis

To generate HIV-1 particles pseudotyped with vesicular stomatitis virus glycoprotein (VSVG), 293T cells seeded in a 10-cm dish at ~70% confluency were transfected with either 7 μg of R7∆Env-GFP or NL4.3Luc-mCherry and 3 μg of pCMV-VSVG using polyethylenimine. Medium was replaced 12 hours after transfection. Viruses were harvested 48 hours after transfection and filtered through a 0.45-μm filter (Millipore). To measure infectivity, equal amounts of NL4.3Luc-mCh or R7-GFP virus, as measured by reverse transcriptase activity, were added to cells, and a synchronized infection was performed by spinoculation at 13°C for 2 hours at 1200*g*. After spinoculation, virus-containing medium was removed and replaced with 37°C medium. Infectivity was measured 48 hours after infection using either luciferase assay to measure presence of firefly luciferase or flow cytometry to measure the number of GFP-positive cells.

#### Proximity ligation assay

A Duolink PLA kit was purchased and assayed per the manufacturer’s protocol. Briefly, cells were grown on coverslips and fixed with 3.7% paraformaldehyde at time points following synchronized infection. To determine interaction between BicDR1 and the viral CA, p24 cells were permeabilized and blocked using 3% donkey serum, followed by incubation with primary antibodies targeting p24 (Santa Cruz Biotechnology, sc-69728) and BicDR1 (Atlas Antibodies, HPA041309). After primary staining, coverslips were washed and incubated (1 hour at 37°C) with secondary anti-mouse conjugated minus and anti-rabbit conjugated plus Duolink PLA probes. Following incubation, coverslips were washed once again and incubated with ligation-ligase solution (30 min at 37°C), followed by subsequent incubation with amplification-polymerase solution (100 min at 37°C) containing Duolink Red reagents. Upon completing the PLA protocol, coverslips were additionally incubated with phosphate-buffered saline (PBS) containing 4′,6-diamidino-2-phenylindole and phalloidin-488 and subsequently mounted.

Z-stack images were acquired using a DeltaVision wide-field fluorescent microscope (Applied Precision, GE Healthcare) equipped with a CoolSNAP camera (CoolSNAP HQ, Photometrics) using a 60× objective lens. Excitation light was generated using an illumination module (Applied Precision, GE Healthcare) and deconvolved using softWoRx deconvolution software. All images were imaged using identical acquisition parameters. Following acquisition, PLA puncta were quantified using Imaris software.

#### Western blots

Cells were harvested and washed with PBS, then lysed on ice for 30 min using passive lysis buffer supplemented with a protease inhibitor cocktail (Pierce). The cell lysates were clarified by centrifugation at 13,000*g* for 15 min. Protein concentrations in the whole-cell lysates were quantified using a bicinchoninic acid protein assay kit (Thermo Fisher Scientific). Equal amounts of protein were heated at 97°C with Laemmli 2× SDS sample buffer for 5 min. The samples were then loaded onto a precast polyacrylamide gel (Mini-Protean TGX, Bio-Rad) for SDS-PAGE and separated by electrophoresis. Proteins were transferred onto nitrocellulose membranes and detected through incubation with primary antibodies: rabbit anti-BicDR1 (Atlas Antibodies, HPA041309), mouse anti-Hook3 (sc-398924), and mouse anti–glyceraldehyde-3-phosphate dehydrogenase (sc-365062), followed by incubation with horseradish peroxidase–conjugated rabbit and mouse secondary antibodies.

### Statistical analyses

All data were analyzed and plotted by Prism 9 or Prism 10. Two-tailed unpaired *t* test with Welch’s correction (did not assume equal SD for both populations) was used to calculate *P* values for pairwise comparison. For non-normal distribution, nonparametric Mann-Whitney *t* test was used. To conduct multiple comparisons, a normality test (based on D’Agostino-Pearson normality test with 0.05 significance level) was first conducted to determine whether the data follow a normal (Gaussian) distribution. If all the datasets followed the normal distribution, then statistical analyses were performed using one-way Brown-Forsythe and Welch analysis of variance (ANOVA) with correction for multiple comparison using the Dunnett’s T3 test. In addition, the nonparametric test was conducted if one or more of the datasets were not normally distributed. Dunn’s test was used to correct for nonparametric ANOVA. Multiple comparisons followed the mean rank of each column with the mean rank of the control column or with every other column. The *P* values are reported on all graphs with asterisks following the GraphPad style with 95% confidence interval: n.s., *P* > 0.05; *P ≤ 0.05; **P ≤ 0.01; ***P ≤ 0.001; ****P ≤ 0.0001.
