# Supplementary material for: HIV-1 binds dynein directly to hijack microtubule transport machinery
Source: Sci Adv. 2025 Jun 18;11(25):eadn6796. doi: 10.1126/sciadv.adn6796 (PMC12175901; doi:10.1126/sciadv.adn6796)
Supplement: Supplementary file 1 — Figs. S1 to S6 [file sciadv.adn6796_sm.pdf]

Science Advances
AAAS

Supplementary Materials for

HIV-1 binds dynein directly to hijack microtubule transport machinery

Somayesadat Badieyan *et al.*

Corresponding author: Michael A. Cianfrocco, mcianfro@umich.edu

Sci. Adv. **11**, eadn6796 (2025)
DOI: 10.1126/sciadv.adn6796

This PDF file includes:

Figs. S1 to S6

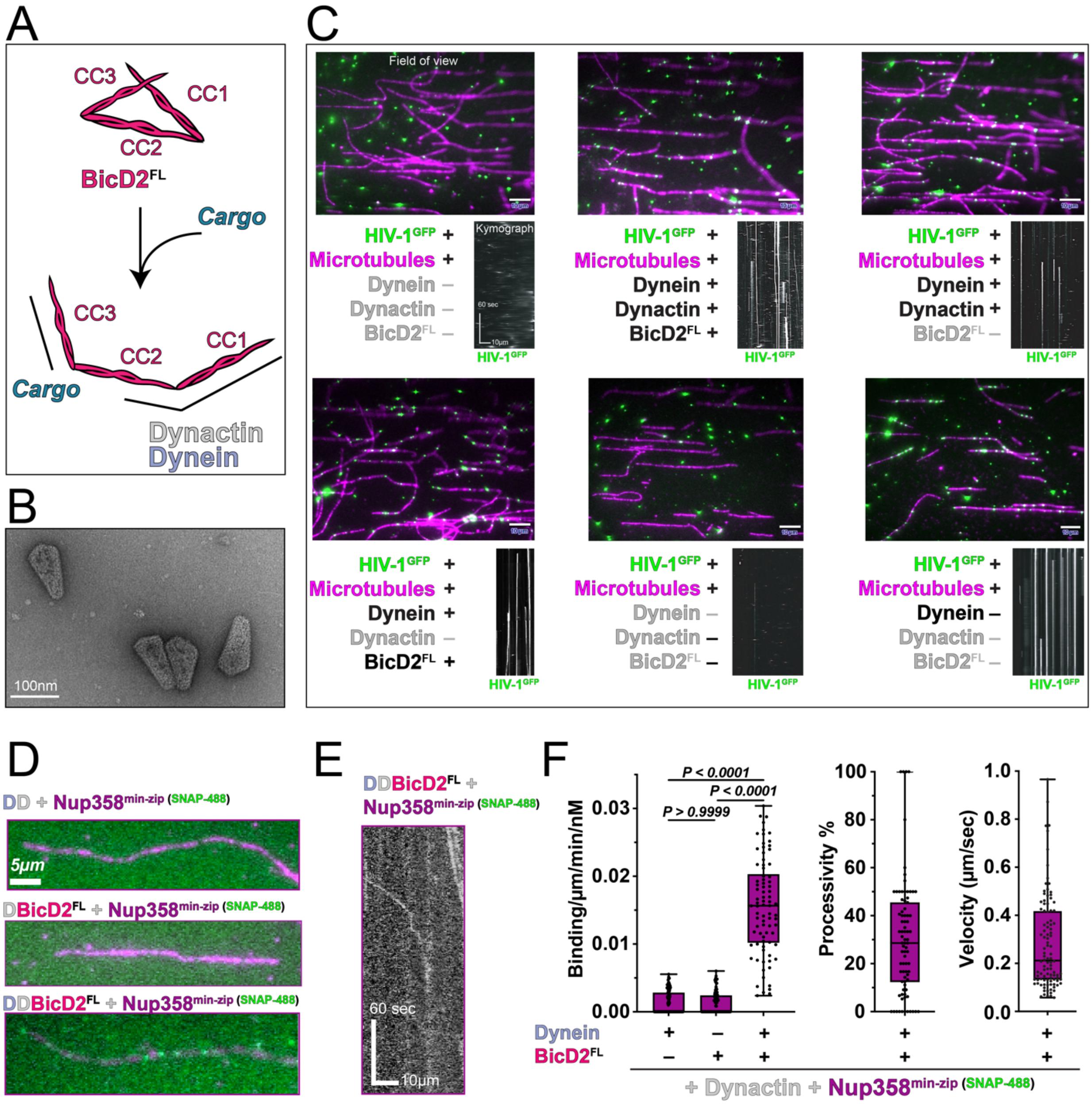

Fig. S1. Supplemental data related to BicD2^{FL} in HIV-1 microtubule recruitment assays.

(A) Proposed model for cargo-mediated activation of BicD2^{FL}. **(B)** Negative staining of purified HIV-1 (GFP-Vpr) cores. **(C)** Field of views and representative kymographs (related to Fig. 1B). Single-molecule assay of HIV-1 (GFP-Vpr) recruitment to microtubules in different combinations of dynein, dynactin, and BicD2^{FL}. **(D-F)** Experimental validation of purified full-length BicD2's capability in recruiting and transporting native cargos: microtubule recruitment assay **(D)**, representative kymograph **(E)**, and related quantifications **(F)** of Nup$^{358min\text{-}zip\ (Snap\text{-}488)}$ with dynein, dynactin, and BicD2^{FL}.

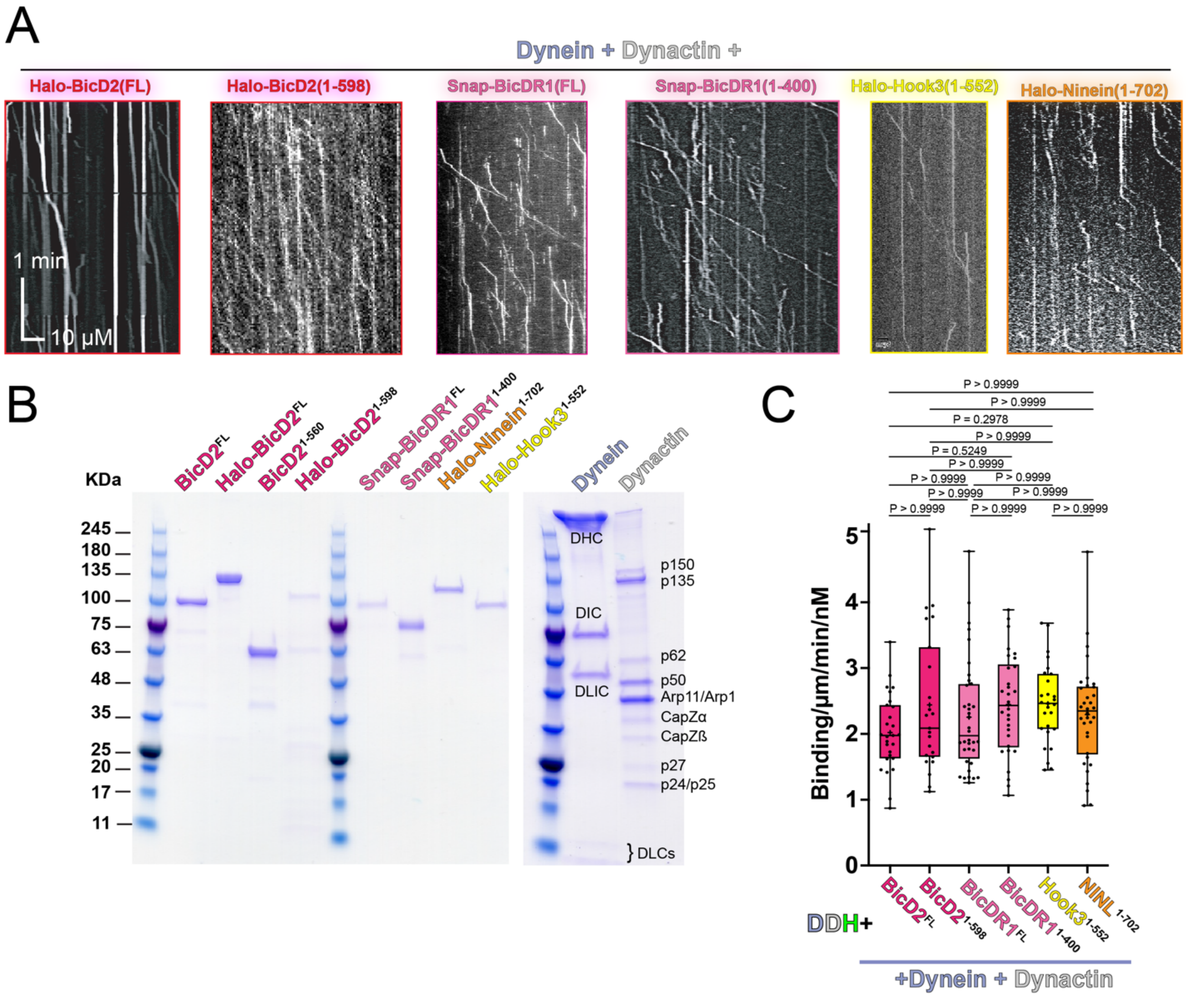

Fig. S2. Motility assay of dynein with different activators.

(A) Motility of dynein-dynactin with indicated adaptor. Dynein and dynactin were unlabeled. Halo-BicD2^{FL}, Halo-BicD2$^{1\text{-}598}$ were labeled by Halo-AlexaFlour 660. Halo-NINL$^{1\text{-}702}$ and Halo-Hook3$^{1\text{-}552}$ were labeled by HaloTag-Alexa Flour 488. SNAP-BicDR1^{FL} and SNAP-BicDR1$^{1\text{-}400}$ were labeled by SnapCell-TMR **(B)** SDS-PAGE of purified activators, dynein and dynactin **(C)** Binding rate of HIV-1 cores in the presence of dynein, dynactin with indicated adaptor.

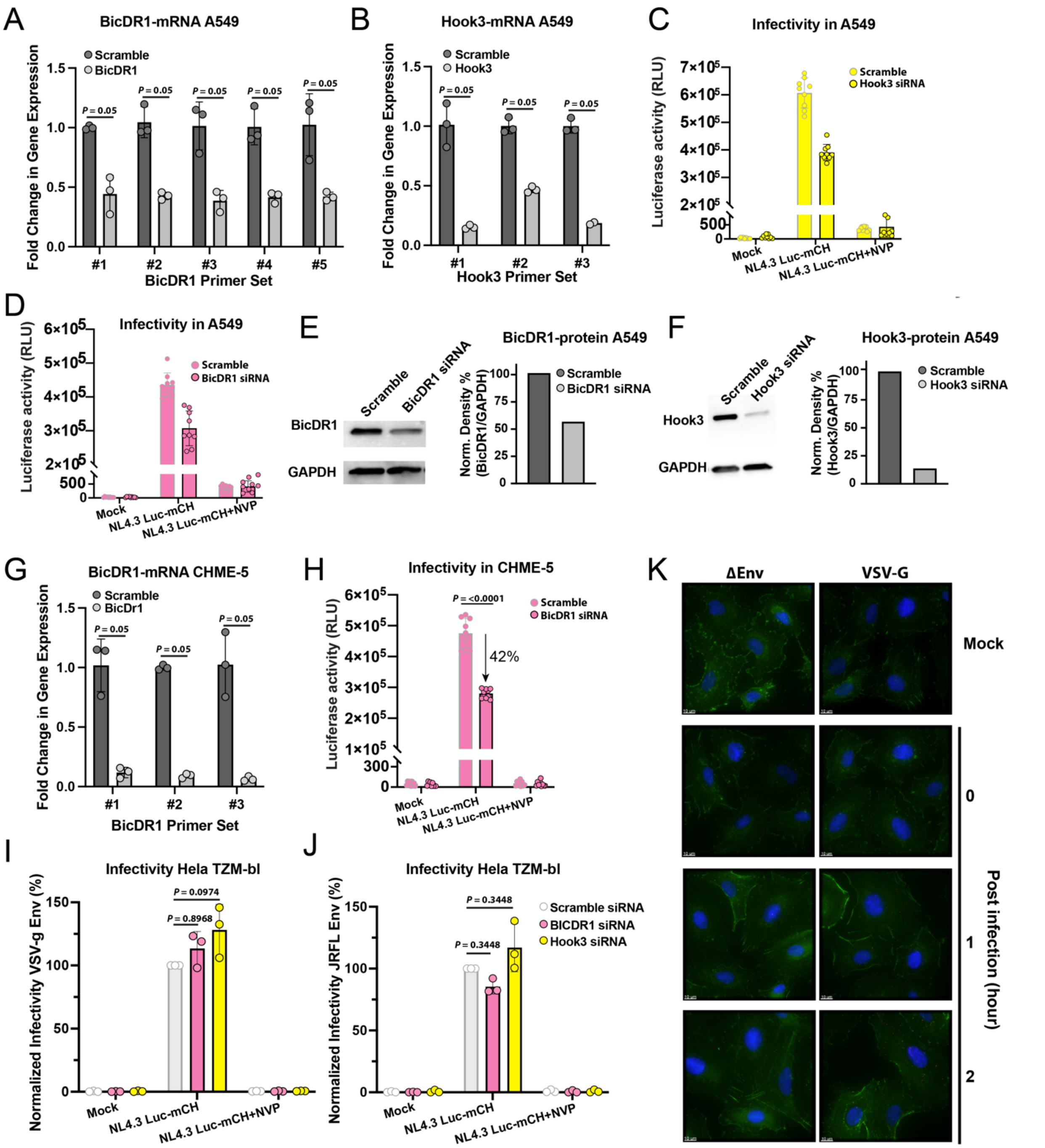

Fig. S3. BicDR1 and Hook3 are required for HIV-1 infection.

(A) BicDR1 mRNA level in BicDR1 depleted A549 cells as tested by qPCR using 5 different primer set. **(B)** Hook3 mRNA level in Hook3 depleted A549 cells as tested by qPCR using 3 different primer set. **(C and D)** A549 cell lacking BicDR1 or Hook3 were infected with NL4.3 Luc-mCH virus pseudotyped with VSV-G envelope glycoprotein. Cell were harvested 48 hours post infection and luciferase activity was measured. Luciferase activity was also measured post-infection when cells were treated by Reverse Transcriptase inhibitor, Nevirapine (NVP). Data

points (±SD) from nine independent measurements are plotted. **(E-F)** Protein expression in A549 BicdR1- or Hook3-depleted cells confirmed by Western blotting and the band densities were quantified by ImageJ. **(G)** BicDR1 mRNA level in BicDR1 depleted CHME-5 cells as tested by qPCR using 3 different primer set. **(H)** CHME-5 cell lacking BicDR1 were infected with NL4.3 Luc-mCH virus pseudotyped with VSV-G envelope glycoprotein. Luciferase activity was measured 48 hours post infection. As a control, infection in presence of Reverse Transcriptase inhibitor, Nevirapine (NVP), was also measured. Data points (±SD) from three independent measurements are plotted. **(I-J)** Normalized infectivity (%) in Hela TZM-bl depleted in BicDR1 or Hook3. The cells were infected with NL4.3 Luc-mCH virus either pseudotyped with VSV-G envelope glycoprotein **(I)** or expressing CCR5 tropic JRFL **(J)**. **(K)** Related to Fig 2G, Proximity Ligation Assay to determine interaction between BICDR1 and the viral capsid protein p24. A549 cell were infected with R7ΔEnv virus pseudotyped with envelope glycoprotein VSV-G. Santa Cruz sc-69728 antibody was used to target p24 and Atlas Antibodies HPA041309 was used to detect BicDR1.

Statistical analyses were performed using non-parametric one-way ANOVA for multiple comparison **(I and J)** and Mann-Whitney non-parametric t-test for pairwise comparison **(C, D and H).**

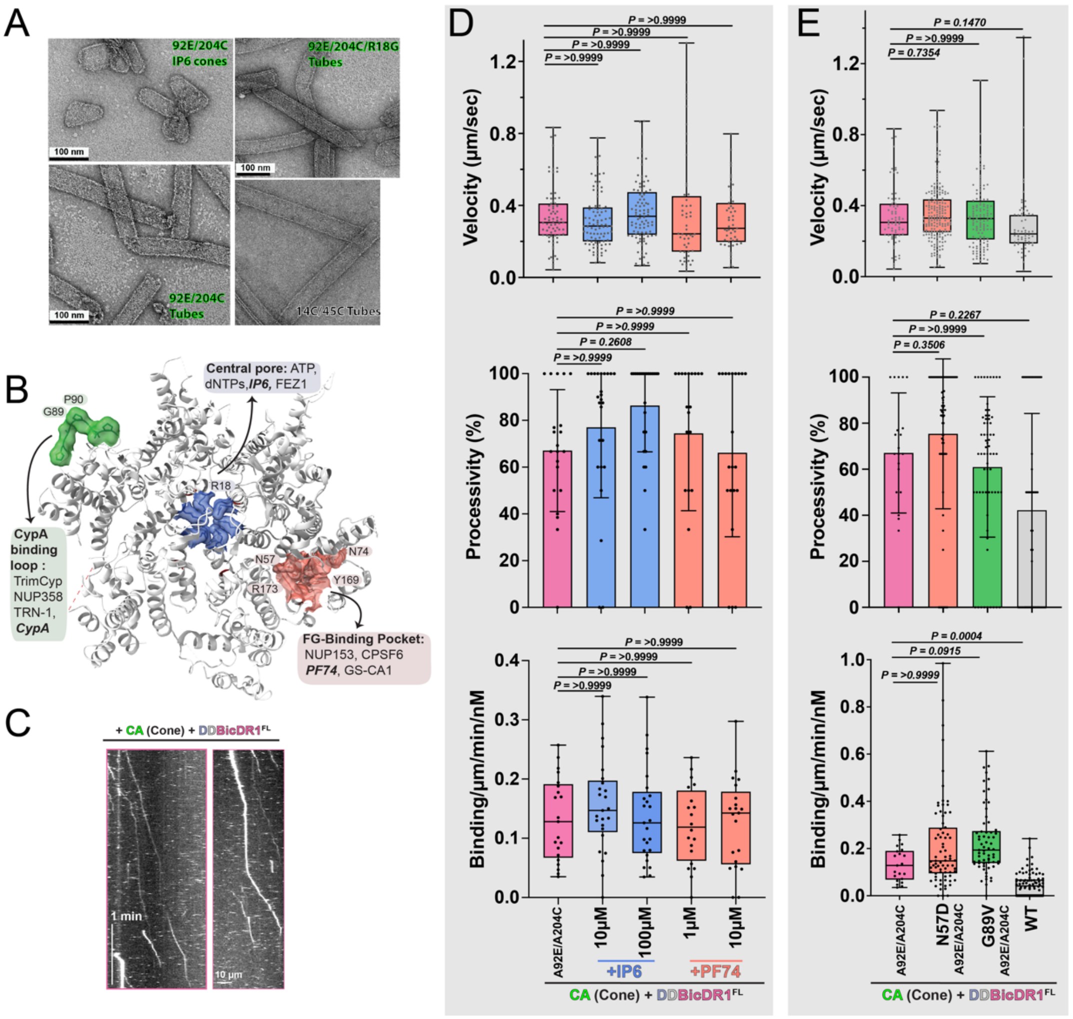

Figure S4 - Competition assay and mutagenesis to determine the binding site of dynein on HIV-1.

(A) Negative staining EM images of cross-linked A92E/A204C cones (as assembled by IP6), cross-linked A92E/A204C or A92E/A204C/R18G or A14C/E45C tube-shaped capsid (as assembled by 1 M salt). A92E/A204C or A92E/A204C/R18G samples are fluorescently labeled by incorporating K157C-CA bound to Alexa-488 into the assembled constructs. **(B)** Three major binding sites for different host factors and small molecules on HIV-1 capsid are presented in a CA-hexamer. **(C)** Representative kymographs of fluorescently labeled HIV-1 A92E/A204C cones trafficking with dynein-dynactin-BicDR1^{FL} (DDR) complex. **(D)** Binding, processivity , and velocity parameters of DDR-mediated motility of CA (A92E/A204C cross-linked cone-shaped capsid assembled by IP6) are plotted in the presence of different concentrations of IP6 and PF-74. The dynein concentration was kept at 10 nM. **(E)** The binding, processivity, and velocity parameters of DDR-mediated motility for wild-type (WT) and mutant cone-shaped capsids are plotted as follows: CA (A92E/A204C), CA (N57D/A92E/A204C), CA (G89V/A92E/A204C) — all cross-linked capsids assembled by IP6, and CA-WT assembled by IP6 without any cross-linking. Imaging of the WT capsid was conducted in the presence of 40 µM IP6.

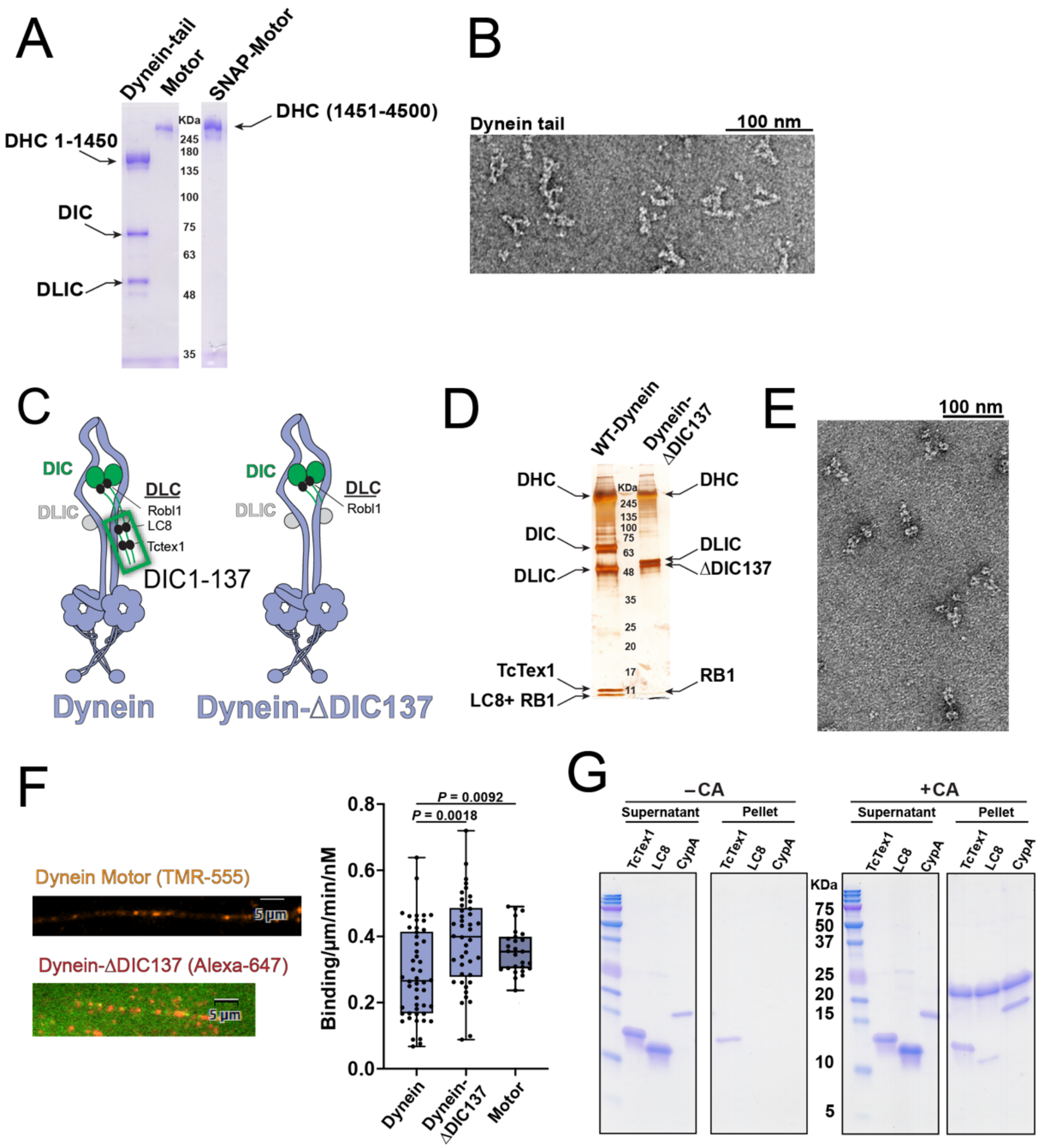

Fig. S5. Validation of dynein motor constructs.

(A) Coomassie-blue staining of SDS-PAGE for purified dynein-tail, motor domain, and SNAP-tagged dynein motor domain. **(B)** Negative staining EM of purified dynein tail. **(C)** Schematic presentation of dynein and dynein-ΔDIC137. **(D)** Silver staining of purified dynein and dynein-ΔDIC137. **(E)** Negative staining of purified Dynein-ΔDIC137. **(F)** Microtubule recruitment assay of TMR-labeled dynein motor and Alexa-647 labeled Dynein-ΔDIC137 and associated quantification of microtubule binding. **(G)** Co-pelleting of CA tubes (14C/45C) with human dynein light chains (TcTex1 and LC8), where CypA is a positive control.

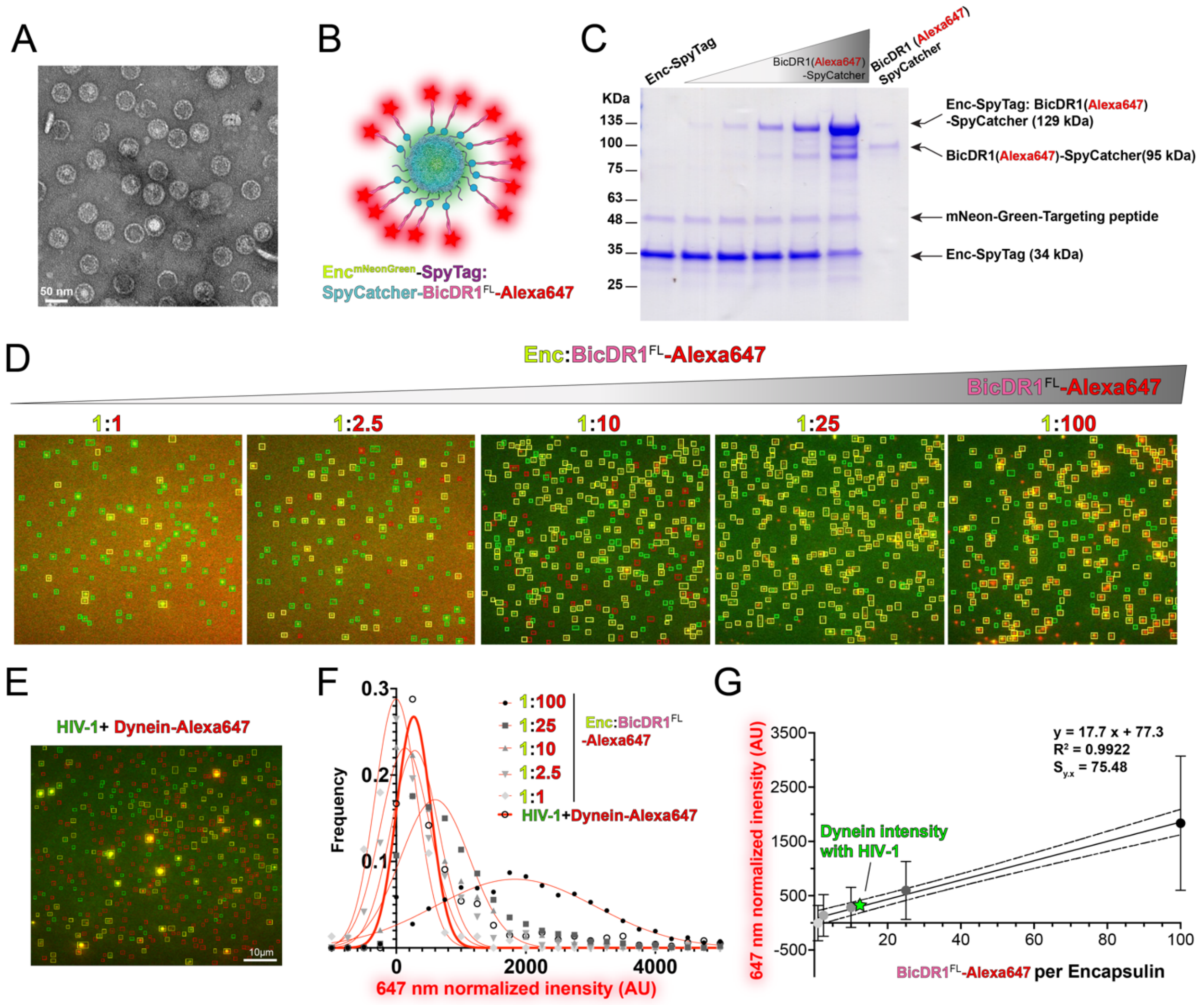

Fig. S6. Dynein quantification on HIV-1 using encapsulin model system.

(A) Negative staining TEM image of T=4 encapsulin (Enc$^{mNeonGreen\text{-}SpyTag}$). **(B)** Model of Encapsulin-SpyTag:SNAP-BicDR1^{FL}-SpyCatcher. **(C)** SDS-PAGE of Encapsulin-SpyTag reaction with BicDR1(Alexa647)-SpyCatcher at different molar ratio of BicDR1(Alexa647)-SpyCatcher. **(D)** TIRF images of encapsulin complexes on coverslip. At each ratio, the 647nm fluorescence intensity that was co-localized with the encapsulin(mNeonGreen) was quantified by ComDet plugin in ImageJ. **(E)** TIRF field of view showing dynein labeled with Alexa 647 on HIV-1 (Vpr-GFP) cores at the saturating ratio of 200 dynein molecules per core. At the same imaging condition as Enc-BicDR1^{FL} (Alexa647), the fluorescence intensity of dynein at 647nm co-localized with HIV-1 core (GFP-Vpr) was measured. **(F)** Histogram distribution of 647nm intensity and fitted Gaussian curve for each Enc-BicDR1^{FL} (Alexa647) construct as

well as for HIV-1 core+Dynein(Alexa 647). **(G)** Full standard curve made out of histogram graph in panel E, and fitted linear regression.
